# Pseudonatural Products Occur Frequently in Biologically Relevant Compounds

**DOI:** 10.1021/acs.jcim.1c01084

**Published:** 2021-10-20

**Authors:** José-Manuel Gally, Axel Pahl, Paul Czodrowski, Herbert Waldmann

**Affiliations:** † Department of Chemical Biology, Max-Planck-Institute of Molecular Physiology, Otto-Hahn-Straße 11, 44227 Dortmund, Germany; ‡ Compound Management and Screening Center, Max-Planck-Institute of Molecular Physiology, Otto-Hahn-Str. 11, 44227 Dortmund, Germany; § Faculty of Chemistry and Chemical Biology, Technical University Dortmund, Otto-Hahn-Straße 6, 44227 Dortmund, Germany

## Abstract

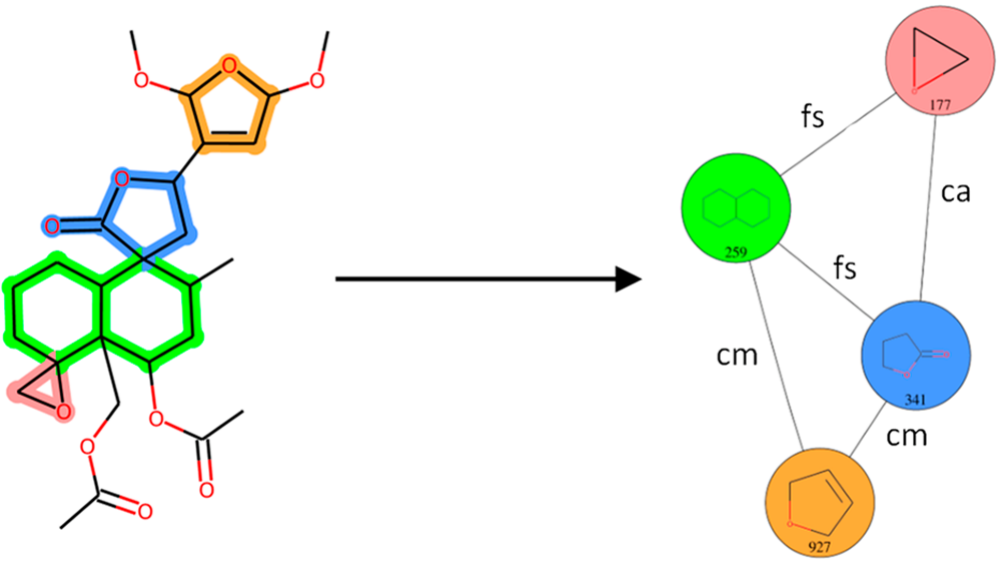

A new methodology for classifying fragment combinations and characterizing pseudonatural products (PNPs) is described. The source code is based on open-source tools and is organized as a Python package. Tasks can be executed individually or within the context of scalable, robust workflows. First, structures are standardized and duplicate entries are filtered out. Then, molecules are probed for the presence of predefined fragments. For molecules with more than one match, fragment combinations are classified. The algorithm considers the pairwise relative position of fragments within the molecule (fused atoms, linkers, intermediary rings), resulting in 18 different possible fragment combination categories. Finally, all combinations for a given molecule are assembled into a fragment combination graph, with fragments as nodes and combination types as edges. This workflow was applied to characterize PNPs in the ChEMBL database via comparison of fragment combination graphs with natural product (NP) references, represented by the Dictionary of Natural Products. The Murcko fragments extracted from 2000 structures previously described were used to define NP fragments. The results indicate that ca. 23% of the biologically relevant compounds listed in ChEMBL comply to the PNP definition and that, therefore, PNPs occur frequently among known biologically relevant small molecules. The majority (>95%) of PNPs contain two to four fragments, mainly (>95%) distributed in five different combination types. These findings may provide guidance for the design of new PNPs.

## Introduction

Natural products (NPs) are a rich source of inspiration for drug discovery, and compounds derived from or inspired by NP structure constitute a major fraction of currently available drugs.^1,2^ In light of this proven relevance, design and synthesis of novel bioactive compounds can benefit from the inclusion of structural properties derived from NPs. We have recently introduced the concept of pseudonatural products^3−5^ (PNPs) as novel NP-inspired compound classes which combine the biological relevance of NPs with the efficient exploration of chemical space by fragment-based compound design.^6^ In PNPs, NP-derived fragments are combined in unprecedented arrangements which are not available by current biosynthesis pathways. They inherit the biological relevance of NPs, yet explore biologically relevant regions of chemical space not accessed by nature, and it can be expected that PNPs may have novel or different bioactivity and targets compared to the guiding NPs.

**Figure 1:**
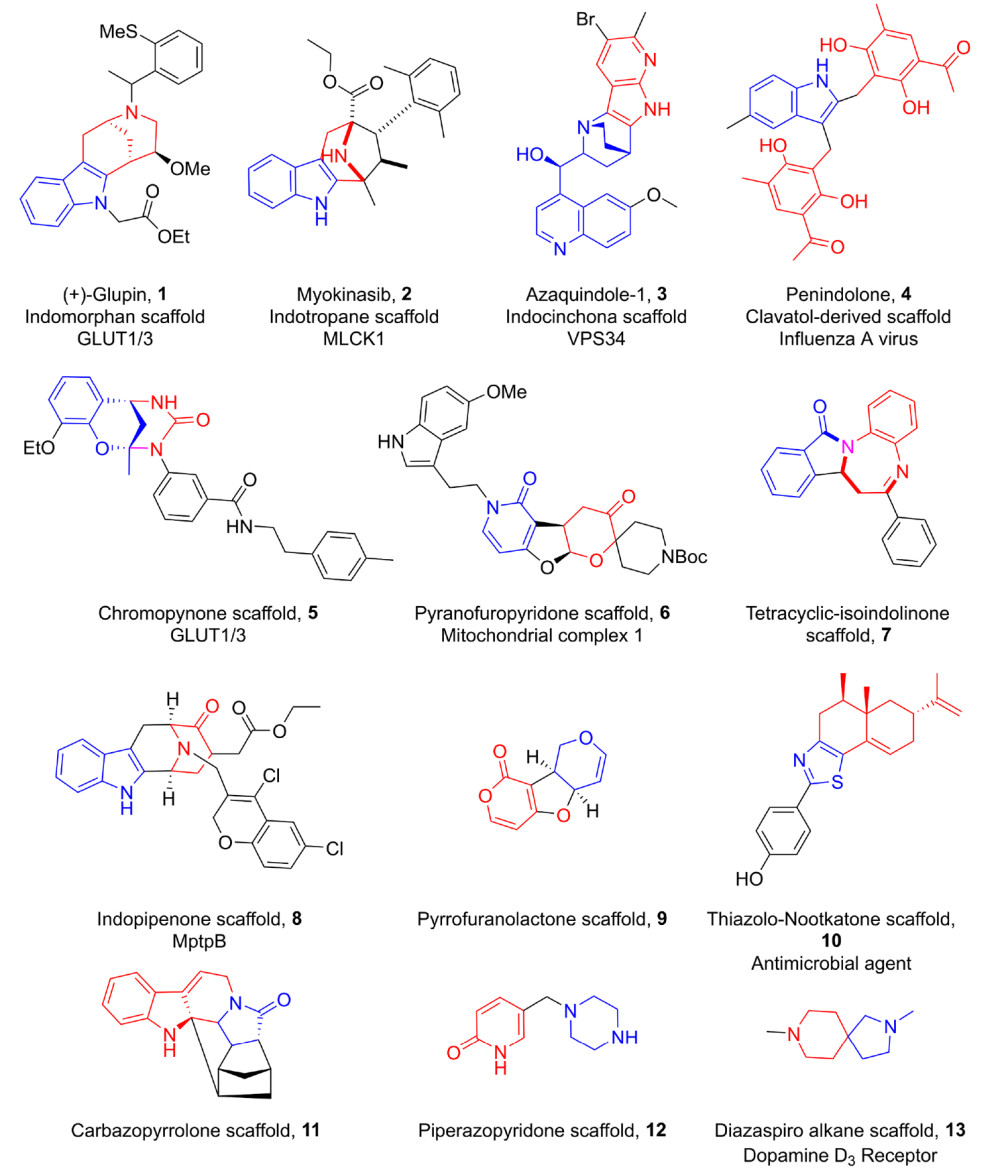
Examples of PNP scaffolds. Red and blue colors denote NP fragments.

Synthesis and biological evaluation of several PNP collections provided proof-of-principle for the concept (Figure 1). For instance, the fusion of indole- with morphan fragments and chromane- with tetrahydropyrimidinone fragments, respectively, resulted in the indomorphan^7^**1** and chromopynone^8^**5** compound classes, which define novel inhibitors of the glucose transporters GLUT-1 and -3. Moreover, fusion of the indole and tropane fragments yielded the indotropane compound class, from which Myokinasib^9^**2**, a MLCK1 inhibitor, could be identified. Another recent PNP class is the indocinchona alkaloids^10^ which are obtained by fusion of indole and cinchona alkaloid fragments. Among this compound class, Azaquindole-1 **3** inhibited the lipid kinase VPS34, thereby suppressing starvation- and rapamycin-induced autophagy. The recombination of pyridine and dihydropyran fragments led to the pyrano-furo-pyridone^11^ PNPs **6**, novel reactive oxygen species inducers and inhibitors of mitochondrial complex I. Li et al. mimicked the biosynthesis of penilactones by synthesis of the PNP penindolone^12^**4**, constituted of an indole and two clavatol fragments. Penindolone showed broad-spectrum anti-influenza A activity. Similarly, Yuan et al. combined the benzodiazepine and isoindolinone scaffolds, found in many drugs and NPs and endowed with broad biological activity, into tetracyclic benzodiazepine-fused isoindolinones **7**.^13^

In addition, cheminformatic analysis revealed that large screening libraries are biased toward biogenic molecules that proteins have evolved to recognize, that is, NPs and related compound classes.^14^ This bias may reflect the historical focus of medicinal chemistry on NPs and the resulting synthesis efforts.

These observations suggest that compound classes that match the PNP definition might have been synthesized and biologically evaluated before, without inspiration by the PNP design principle, for instance, driven by intuitive inclusion of different NP structures in medicinal chemistry and chemical biology synthesis programs (Figure 1).

For instance, the cycloocta[*b*]indole compound class,^15^ which was designed and synthesized following the biology-oriented synthesis principle based on the NP macroline and which targets the mycobacterial phosphatase MptpB, could be described as the result of the fusion of indole and piperidone fragments. Therefore, in hindsight it was termed indopipenone^3^**8**. Other examples include the carbazopyrrolone^16^**11** and pyrrofuranolactone^17^**9** compound classes. In some cases, bioactivity could be detected, for example, for piperazopyridones^18^**12** (TRPV6 calcium channel inhibitors), diazaspiro alkanes^19^**13** (dopamine D_3_ antagonists), and thiazolo-nootkatones^20^**10** (antimicrobial agents).

Hence, PNPs might already constitute a larger fraction of currently available and applied bioactive small molecules. They might have proven to be endowed with biological relevance in general, to display diverse bioactivity, and to constitute already widely explored NP inspired chemotypes in chemical biology and medicinal chemistry research and drug discovery. Such wide application and exploration would validate the PNP principle in a general sense.

In order to explore this possibility, we have analyzed the ChEMBL^21^ database, which lists biologically relevant small molecules including their structure and activity, for compounds that conform to the PNP definition. We report that ca. 23% of the biologically relevant compounds listed in ChEMBL and considered in the analysis can be classified as PNPs and that, therefore, PNPs occur frequently among known biologically relevant small molecules. Based on our analysis, we conclude that the majority (>95%) of PNPs in ChEMBL represent combinations of two to four fragments and five fragment combination types. This finding may provide guidance for the design of new PNPs.

## Results and Discussion

**Figure 2:**
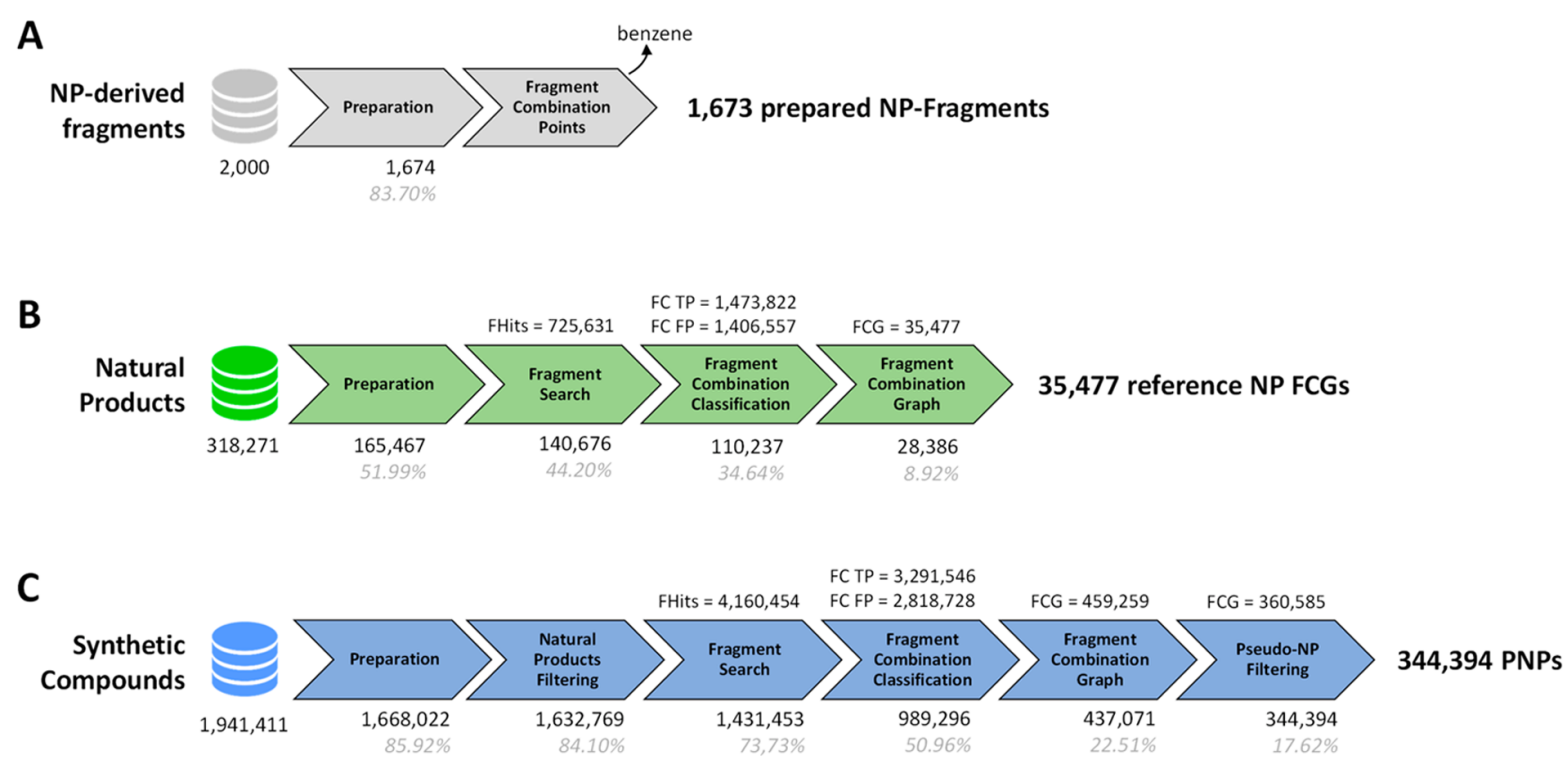
Workflows applied to the different data sets. (A) The workflow for fragments. (B) The workflow for NPs (DNP). (B) The workflow for synthetic compounds (ChEMBL). The number of remaining molecules at each step is displayed below the tasks when changes occur. Below it, the percentage of remaining molecules in regards to the initial number is displayed in gray. Above the tasks, the number of observed elements is displayed when different from molecules. FHits, fragment hits; FC, fragment combinations; TP, true positive; FP, false positive; FCG, fragment combination graphs.

For identification and analysis of the NP fragments and NP fragment combinations, we established an analysis package written in Python 3 and termed it natural product fragment combination (NPFC). NPFC consists of a set of modules and scripts and employs the open-source libraries RDKit^22^ (v. 2020.09.1) for processing chemical structures, Pandas^23^ (v. 1.1.4) for data handling, Networkx^24^ (v. 2.5) for representing fragment connectivity as graphs, and Snakemake^25^ (v. 5.27.4), for coordinating the different tasks as workflows. The modules and scripts comprise preparation steps including different filtering operations, fragment search, combination and classification steps, establishment of fragment combination graphs, and finally PNP annotation (Figure 2).

For the analysis, three different data sets were employed, that is, NP fragments to be used in the fragment search, data for NPs, and the fragment combinations in them and synthetic non-natural compounds listed in ChEMBL to be analyzed for fragment and fragment combination content.

### Preparation of the Data Sets

**Figure 3:**
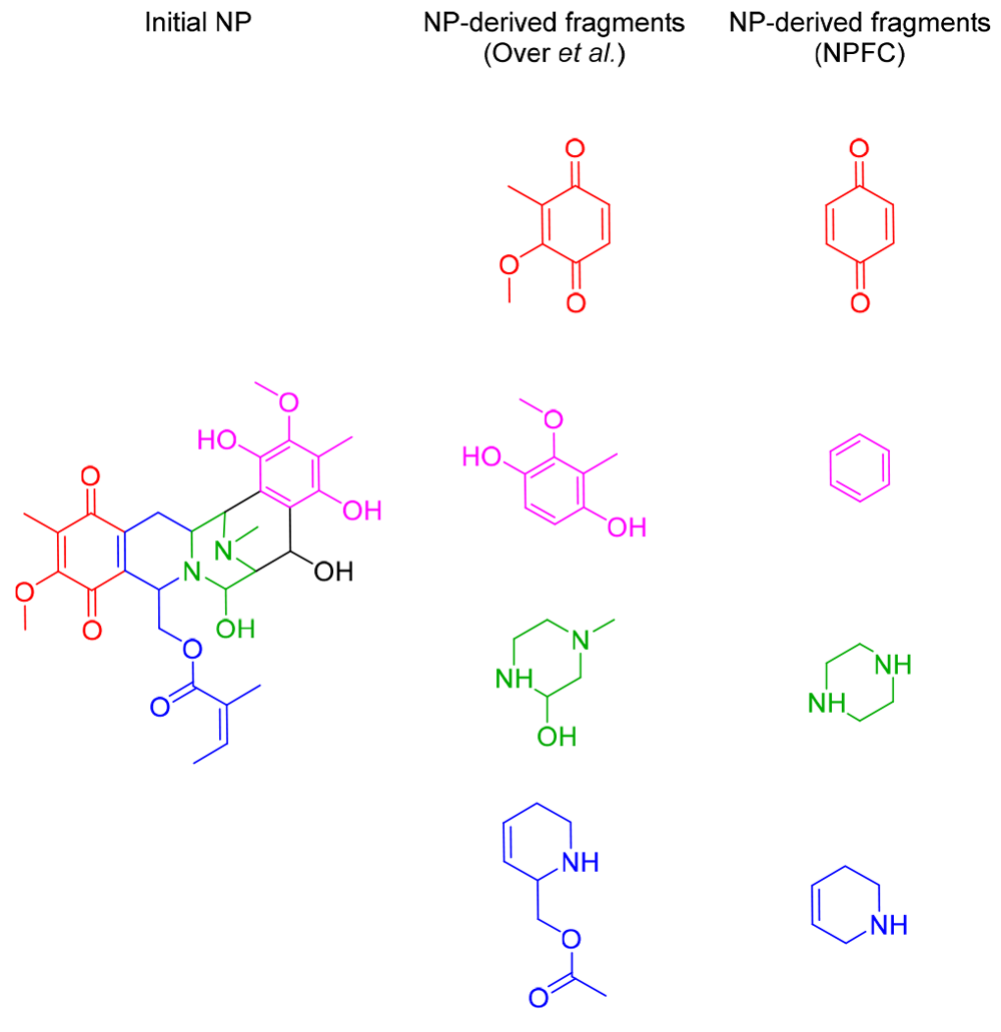
NP-derived fragments. The structures generated by Over et al.^26^ were used as a basis for defining the NP-derived fragments used here. The benzene fragment was removed from the prepared NP-fragment data set.

For the identification of NP fragments, we employed a set of structurally diverse and biologically relevant 2000 NP-derived fragments, obtained through cycles of side chain pruning and ring degeneration, as described by Over et al.^26^ (Figure 3), and available in SDF format. The processing of fragments consisted in two main steps: the preparation of the data set (load, standardize, deduplicate, see below) and the annotation of fragment combination points. Five structures in the data set could not be parsed and converted to the RDKit format and were manually curated (see the Supporting Information).

Subsequently, the input file was loaded without errors into a Pandas DataFrame with structures in RDKit format. Next, a set of sequential tasks was applied to the structures for standardization. First, records with empty structures are filtered out. Mixtures are cleared, preferring the largest, nonlinear organic compound possible. Structures were further altered using the functionalities of MolVS^27^ as implemented within RDKit. Thus, atoms were set to the most common isotope only, functional groups were normalized in their representation (e.g., charge-separate nitro groups), formal charges were removed whenever possible, the canonical tautomer was enumerated, and stereochemistry information was removed. Finally, Murcko scaffolds were extracted from the structures, followed by another round of removing formal charges. None of these steps resulted in the elimination of compounds from the data set.

Duplicate structures were then filtered out using InChI Key as identity, decreasing the size of the data set to 1673 entries (84% of the initial data set). To obtain the best possible depiction for each structure, different methods available in the RDKit were applied to generate 2D coordinates (CoordGen and rdDepictor). A third-party library^28^ was employed to score the depictions based on their number of overlapping bonds and atoms and to keep the depiction with the lowest score. Input coordinates were considered as well, when available.

Finally, to identify redundant fragment orientations in combinations, symmetry classes within the structures were annotated as fragment combination points by performing a fragment search of each fragment within itself.^29^

To define the NP chemical space, the Dictionary of Natural Products^30^ (DNP, 318,271 records), which is the result of a comprehensive curation and integration of NP structures with known biological origin, was consulted. The corresponding input SDF had first to be converted to UNIX format using the dos2unix utility, before it could be processed. Stereochemical information was absent from the structures provided by the supplier, therefore the analysis was performed without regarding stereochemical information.

To scale up the computation on a cluster, the input SDF was split into chunks of 5000 entries, for a total of 64 chunks, which were then processed almost completely independently. Molecules were converted to RDKit format with minimal losses (0.13% of the initial data set), with most errors due to incompatibility in aromaticity perception by the RDKit.

Structures were standardized with the following procedure. First, empty structures were removed (9.15% of the initial data set). Then, metal atoms were disconnected from the structures, and organic nonlinear minor compounds were extracted from mixtures, when applicable. To remove sugar units in NPs,^31^ an in-house script was developed using the RDKit, to detect sugar-like rings and peel them off iteratively, starting from the outer layer on the molecule. A set of filters was then applied to remove unwanted entries, based on the number of heavy atoms (*x* ≥ 4, 0.03%), molecular weight (*x* ≤ 1000.0 Da, 1.25%), number of rings (*x* ≥ 1, 7.26%), and chemical elements (only authorized: H, B, C, N, O, F, P, S, Cl, Br, I, 0.08%). Since the structures were altered from earlier operations, they were explicitly sanitized to update the various computational properties of atoms and bonds and possibly avoid errors downstream. Isotopes were then set to their default, the most occurring form, and functional groups were normalized. Then, charges were removed on molecules wherever possible, canonical tautomers enumerated, and, for consistency, stereochemical information removed when applicable. Finally, the structures were regenerated from SMILES. To avoid longer computational times, due to only a small fraction of problematic structures in the data set, a timeout was set to 10 s per molecule for the entire standardization (1.43% of the data was removed by the timeout).

For deduplication, a common reference file was used for all chunks. This reference file contained the list of all already processed compounds, identified by InChI keys. A lock system was applied to ensure that this file could be accessed (read and write) by only one chunk at the time. This method allowed each chunk to safely filter out duplicate structures contained by itself as well as duplicate structures found in other chunks. Since the stereochemistry was not considered, a large portion of the data set (28.67%) was found to be duplicates and was therefore filtered out, further decreasing the size of the data set to 165,467 records (52% of the initial data set).

To define synthetic compounds, the ChEMBL data set was downloaded from the official Web site^32^ as a single SDF of 1,941,411 structures, then divided in 389 chunks of 5000 records. For consistency, the exact same preparation protocol as described above for the DNP was applied, which decreased the total number of entries to 1,668,022 (85.92%), mainly due to filters: duplicates (9.25%), molecular weight (1.70%), timeout (1.21%), and number of rings (1.06%).

Additionally, NPs were removed from the ChEMBL data set to better differentiate NPs from synthetic compounds. To achieve this, duplicate structures of DNP inside of ChEMBL were filtered out by means of InChI Key comparison, decreasing the synthetic data set to 1,632,769 (84.10%) records.

### Fragment Search

A substructure search was performed to identify all fragments occurring in the NPs (fragment hits). Initial results showed an abundance of the benzene fragment, accounting for, respectively, 16% and 38% of all fragments hits in DNP and ChEMBL. This high prevalence, in particular for ChEMBL, reflected in subsequent results, introduced a bias in our conclusions (see the Supporting Information for complete results). Benzene was therefore removed from the fragment pool and was not further considered in this study.

Following this procedure, 140,676 molecules (85% of the remaining molecules) were found to contain at least one NP fragment, for a total of 725,631 fragment hits. This high proportion observed for NPs containing NP fragments was expected, especially since the fragments originated from an earlier version of the same database (DNP 18.2).^26^

As for the NPs, the benzene was also removed from the pool of fragments used for the fragment search in ChEMBL which resulted in 4,160,454 fragment hits, for a total of 1,431,453 compounds (73.73% of the initial data set).

### Fragment Combination

The initial classification proposed by Karageorgis et al.^7^ introduced five different connectivity types, that is, monopodal and bipodal connections as well as spiro, edge, and bridged fusions. To better capture the complexity of NP fragment arrangements, we extended this classification and considered up to 18 fragment combinations and grouped them in categories, types, and subtypes, when applicable. For this step, only the fragment atoms found in rings were considered for the classification of combinations. For each molecule, all possible fragment pairs were investigated independently.

**Figure 4:**
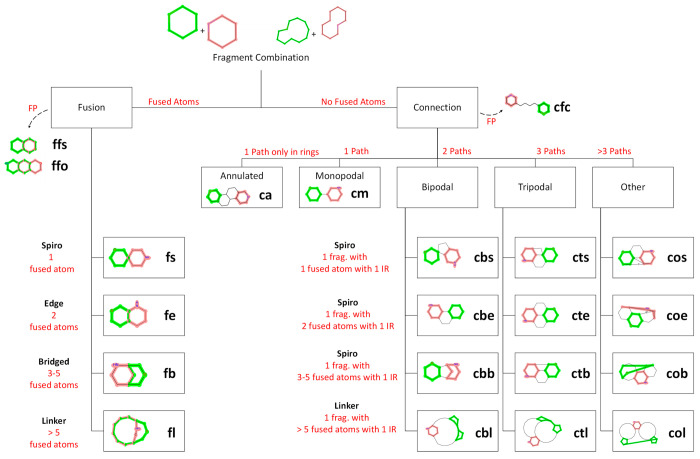
The decision tree applied for classification of fragment combinations. Fragments are highlighted in red and green; fused atoms and bonds are highlighted in both red and green; and intermediary ring (IR) is defined as a ring directly located between both fragments.

The first step of our algorithm for classifying fragment combinations was to identify atoms involved in a fusion between both fragments. If any combination was found, then the combination category was defined as “fusion”. The number of fused atoms then determined the type of fusion (see Figure 4 for a graphical depiction):

*n* = 1 fused atom: spiro
*n* = 2 fused atoms: edge
3 ≤ *n* ≤ 5 fused atoms: bridged
*n* > 5 fused atoms: linker

This resulted in four classes of fusions: fusion spiro (**fs**), fusion edge (**fe**), fusion bridged (**fb**), and fusion linker (**fl**).

The combinations of fragments that did not have any fused atoms were categorized as connections. Intermediary rings between both fragments were defined as rings in the molecule that contained fused atoms with both fragments. The number of identified intermediary rings determined the number of distinct paths that led one fragment to another. This number defined the degree of connection of the combination:

*n* = 1 path: monopodal (no intermediary ring)
*n* = 2 paths: bipodal (1 intermediary ring)
*n* = 3 paths: tripodal (2 intermediary rings)
*n* > 3 paths: other (>2 intermediary rings)

For instance, a bipodal connection could be described as two fragments having no fused atoms with each other but sharing one intermediary ring between them.

A subtype was defined for bipodal connections and of higher degree as well. For each intermediary ring, the atoms from each fragment that were also present in the intermediary ring constituted the fragment connection points (CP). Hence, the number of CPs indicated the interface exposure of each fragment with the intermediary ring considered. For consistency, the same nomenclature used for the type of fusions was applied:

*n* = 1 CP for any of the two fragments: spiro
*n* = 2 CP for both fragments: edge
3 ≤ *n* ≤ 5 CP for any of the two fragments: bridged
*n* > 5 CP for any of the two fragments: linker

Since there could be multiple intermediary rings, several subtypes could be available. Thus, a priority had to be set to decide the subtype of the connection, considering all intermediary rings. The following order was retained to highlight less common fragment combinations: linker > spiro > bridged > edge.

For fragment combinations with no intermediary rings, a distinction was made between monopodal connections (fragments connected through a linker) and annulated connections, where both fragments belonged to the same ring complex but were separated by more than one ring.

In total, 14 fragment combinations (see Figure 4) were found to be connections: connection monopodal (**cm**), connection annulated (**ca**), connection bipodal spiro (**cbs**), connection bipodal edge (**cbe**), connection bipodal bridged (**cbb**), connection bipodal linker (**cbl**), connection tripodal spiro (**cts**), connection tripodal edge (**cte**), connection tripodal bridged (**ctb**), connection tripodal linker (**ctl**), connection other spiro (**cos**), connection other edge (**coe**), connection other bridged (**cob**), and connection other linker (**col**).

In addition to the 18 fragment combination categories described above (fusions and connections), three cases were considered to be false positives. The first class of false positives occurred when two fragments were too far apart from each other in the molecule. A maximum distance threshold, for considering two fragments to have a meaningful combination, was set to 3 intermediary atoms between both fragments. Any combination with more intermediary atoms than the threshold was systematically ignored (connection false positive cut off, **cfc**). These combinations were directly filtered out during classification and were therefore not included in the count of false positives at the end of the computation.

The second class of false positives concerned fragment inclusion. If one fragment completely contained another, then the other fragment was simply a substructure of the first fragment (fusion false positive substructure, **ffs**). In this case, only the larger fragment was retained for the analysis.

The third class of false positives was found for fragment hits with a large proportion of atoms in common. The results indicated that they were not actually fused by synthesis design, but rather overlapping as an artifact of the fragment search. To identify such cases, an arbitrary rule was established that if the atoms in common between the two fragments constituted a full ring in the molecule, then it was not considered to be a proper fusion, but rather an overlap of the fragments (fusion false positive overlap, **ffo**).

Finally, molecules with at least one valid combination were kept at the end of this step. For NPs, this step amounted to 110,237 remaining NPs accounting for a total of 2,880,379 fragment combinations. Half of those combinations (48.84%) were actually false positives (**ffs** or **ffo**) and the vast majority of the remaining NPs contained at least one **ffs** combination (84.72%), up to a maximum of 14, whereas about half (47.63%) contained at least one **ffo** combination (up to 9). Only 12.07% of the NPs at that stage had no false positive combinations.

For ChEMBL, this step resulted in 989,296 molecules (50.96% of the initial data set), accounting for 6,110,274 fragment combinations. Also, in this case, only less than half of the identified combinations (46.13%) constituted valid fragment combinations. The majority of synthetic compounds (68.67%) contained at least one false positive combination, with **ffs** combinations being much more represented than **ffo** combinations (respectively 66.55% and 13.76% of remaining compounds having at least one of those).

### Fragment Combination Graph

To represent the entire fragment connectivity, fragment combinations were assembled into fragment combination graphs (FCG), with fragment types (represented by fragment ids) as nodes (i.e., frag 1, 258, etc.) and combinations as edges (cm, fe, etc.).

In case of false positive combinations **ffs** (one fragment was a substructure of another), only the larger fragment was considered for the analysis, hence avoiding redundant edges. Moreover, fragment graphs that contained overlapping fragments were considered to represent alternative fragment connectivities and were therefore split up into different FCGs. To avoid a combinatorial explosion of the number of graphs due to overlaps, a maximum threshold of five overlaps (**ffo** combinations) was set per molecule. Structures above this threshold were filtered out. In addition, graphs containing disconnected subgraphs were separated into new entries as well.

Only graphs containing at least one valid fragment combination were further considered, decreasing the final number of NPs in the data set to 28,386 structures (8.92% of the initial size), for a total of 35,477 fragment combination graphs.

For ChEMBL, the assembly of FCGs resulted in further reduction of the data set size by half (437,071 structures, 22.51% of the initial data set). The relatively low amount of **ffo** combinations resulted in most molecules containing only 1 FCG (86.24%), for a total of 459,259 graphs.

### PNP Filtering

A PNP was defined as a synthetic compound with a NP fragment connectivity not found in any known NP. Hence, each FCG of a probed molecule was compared with each graph of every NP. In case that the probed molecule’s FCG differed from each NP graph by at least one fragment combination, the FCG was labeled as PNP. In contrast, if an NP graph is found to contain the same fragment combinations as the probed molecule graph, the latter was labeled as NP-like because it represents fragment combinations found in a NP. This can lead to a situation that one molecule can contain graphs with PNP- and NP-like character at the same time. If at least one graph of the probed molecule was labeled as PNP, then the molecule was identified as a PNP. Otherwise, it was labeled as NP-like.

To prioritize innovative fragment combinations rather than repetitions, the number of occurrences of a fragment combination was not considered during comparison. Indeed, a molecule with three repeated fragment combinations would be matched with a molecule containing only one of the same fragment combinations.

**Figure 5:**
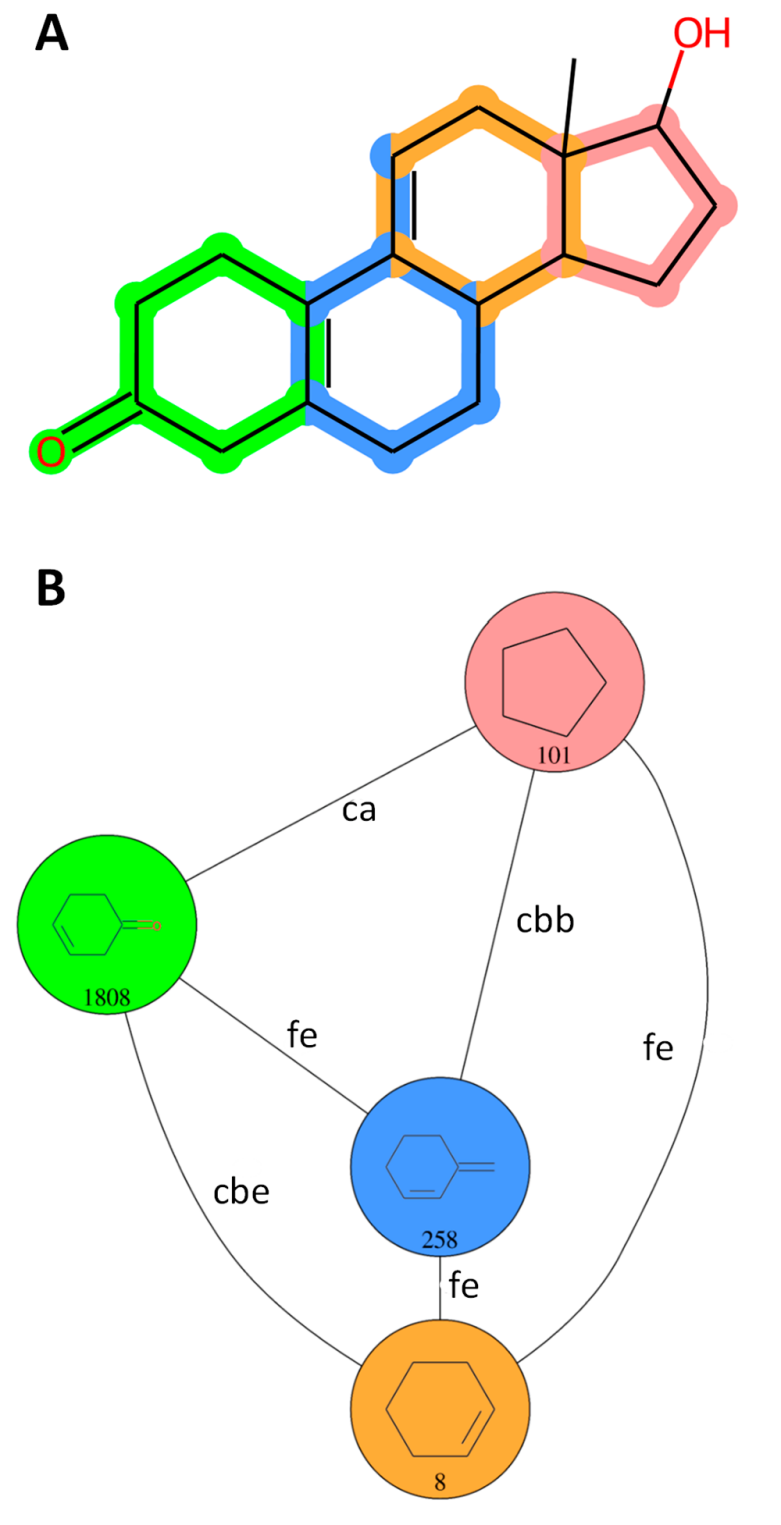
Example of a PNP (CHEMBL2311179). (A) Structure with highlighted NP-derived fragments. Fused atoms and bonds are highlighted with both colors of each fragment. (B) Corresponding fragment combination graph, with nodes annotated with fragment structures and IDs and edges annotated with fragment combination classes.

The pairwise comparison of the 459,259 FCGs from ChEMBL with each of the 35,477 FCGs from the DNP resulted in the identification of 344,394 PNPs (example in Figure 5). This accounted for 78.80% of the remaining synthetic compounds, that is, containing at least two NP-derived fragments involved in at least one meaningful combination, and 17.62% of the whole ChEMBL data set.

### Comparison of NPs and PNPs

The connectivity of most PNP NP fragments could be captured with only one FCG per molecule (1.05 on average), whereas more FCGs per molecules were required for NPs (1.25 on average). This observation was consistent with NPs having proportionally three times more **ffo** combinations than synthetic compounds and a fortiori PNPs. For analyzing results, all FCGs of a molecule were merged into one, while common parts where only considered once.

**Figure 6:**
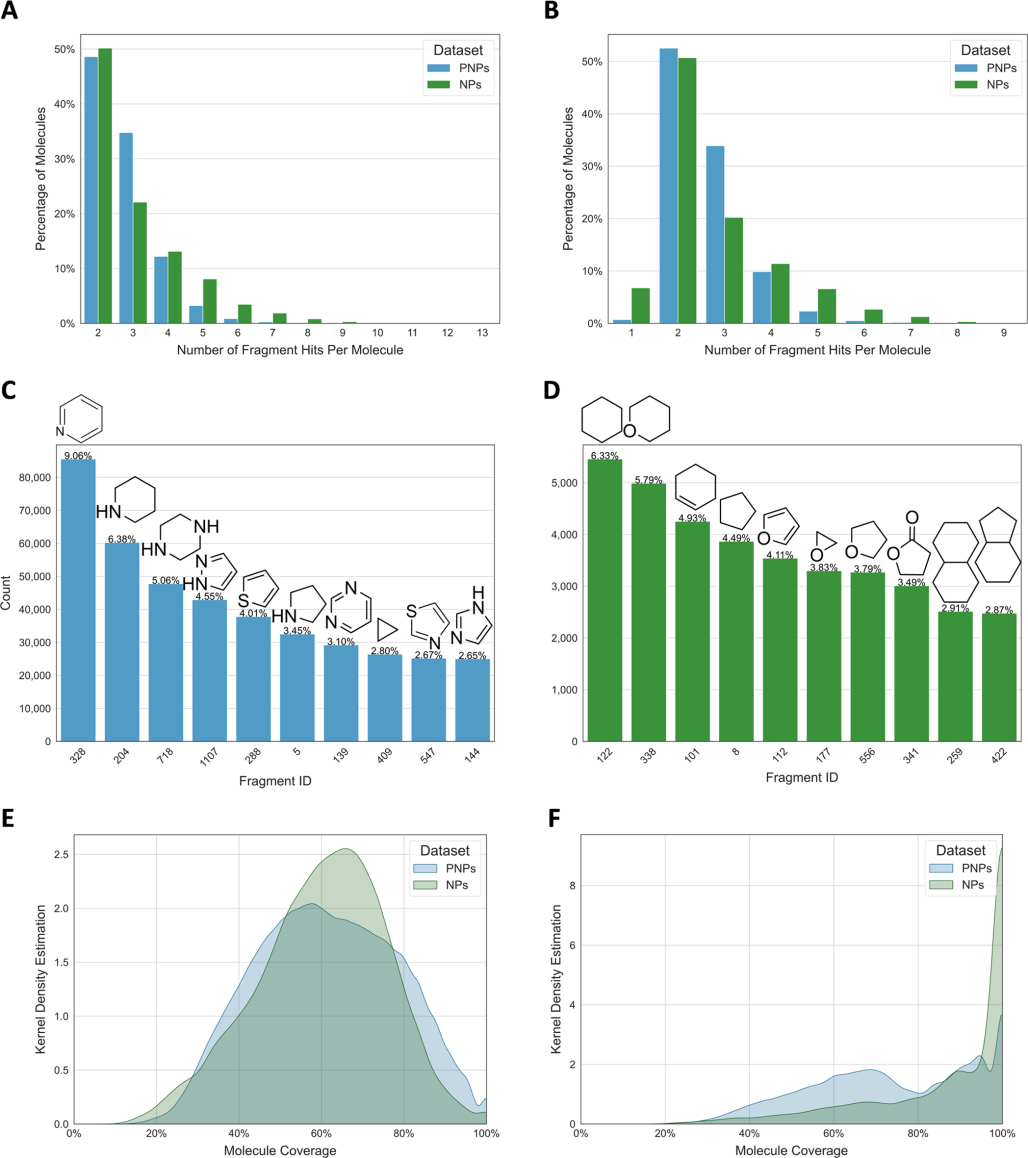
Comparison of PNPs and NPs. (A) Number of fragments per molecule. (B) Number of unique fragments per molecule (each fragment type is counted only once). (C) Ten most occurring fragments in PNPs. (D) Ten most frequently occurring fragments in NPs. (E) Molecule coverage by fragments per molecule, defined as the ratio of the number of heavy atoms found in fragments to the total number of heavy atoms of the molecule. (F) Molecule coverage by fragment per molecule, while considering only rings and linkers in molecules.

The large majority of PNPs (95.58%) contains 2–4 NP fragments, whereas this number ranged more widely from 2 to 7 fragments for NPs (98.77%, Figure 6A). These values did not vary for PNPs, when considering every fragment type only once per molecule (96.29% had 2–4 fragments, Figure 6B). The impact on NPs was much more noticeable, with 99.59% having 1–7 fragments, indicating that NPs had more repeated fragments than PNPs. The number of occurrences of each fragment type was measured for both data sets (1.05 ± 0.18 for PNPs and 1.15 ± 0.41 for NPs) and validated this assumption.

The 10 most abundant fragments observed in PNPs (Figure 6C) accounted for 43.72% of all fragment hits for that data set and were mostly (7 of 10) constituted of nitrogen-containing single rings, with half of them being aromatic. For NPs (Figure 6D), the top 10 fragments represented 42.55% of all fragment hits and consisted mostly (9 of 10) in nonaromatic rings. Half of the most occurring fragments contained only carbon, and the only heteroatom was oxygen.

To assess how much of the molecular topology was captured with our fragment connectivity description, the molecule coverage by fragments was calculated as the percentage of heavy atoms found in fragments compared to the total number of heavy atoms of the molecule (Figure 6E). The molecule coverage by fragments was very similar for PNPs and NPs (61% ± 17 and 60% ± 16, respectively, on average, with standard deviations as margins). The distribution shape of both data sets was further analyzed using the SciPy^33^ library (see the Supporting Information). The distribution shape for both NPs and PNPs was found to be bimodal, with a main peak (at 66% and 58%, respectively) followed by a smaller one at 100%. Although the distribution shape looked in both cases mostly symmetrical and bell-shaped, the distributions were not found to be Gaussian. Since NPs frequently have multiple substituents and side chains, we computed the molecule coverage while considering only rings and linkers in molecules (Figure 6F). This resulted in an increase of the molecule coverage for PNPs (74% ± 19) and to a stronger extent for NPs (88% ± 17). The distribution shapes are very different for both data sets. For NPs, the shape showed two small peaks at 68% and 90%, followed by a very high peak at 100%. The computation of kurtosis and skew indicated that the distribution was significantly more peaked than is to be expected from a normal distribution (kurtosis = 1.61) and was significantly skewed to the left (skew = −1.54). For PNPs, three peaks were also observed at 69%, 95%, and 100%. Although the latter was the most peaked of all, the difference was not as important as for NPs. The distribution was found to be significantly less skewed as well (skew = −0.30) and overall slightly flatter than a Gaussian distribution (kurtosis = −0.96). No distribution was found to be Gaussian.

**Figure 7:**
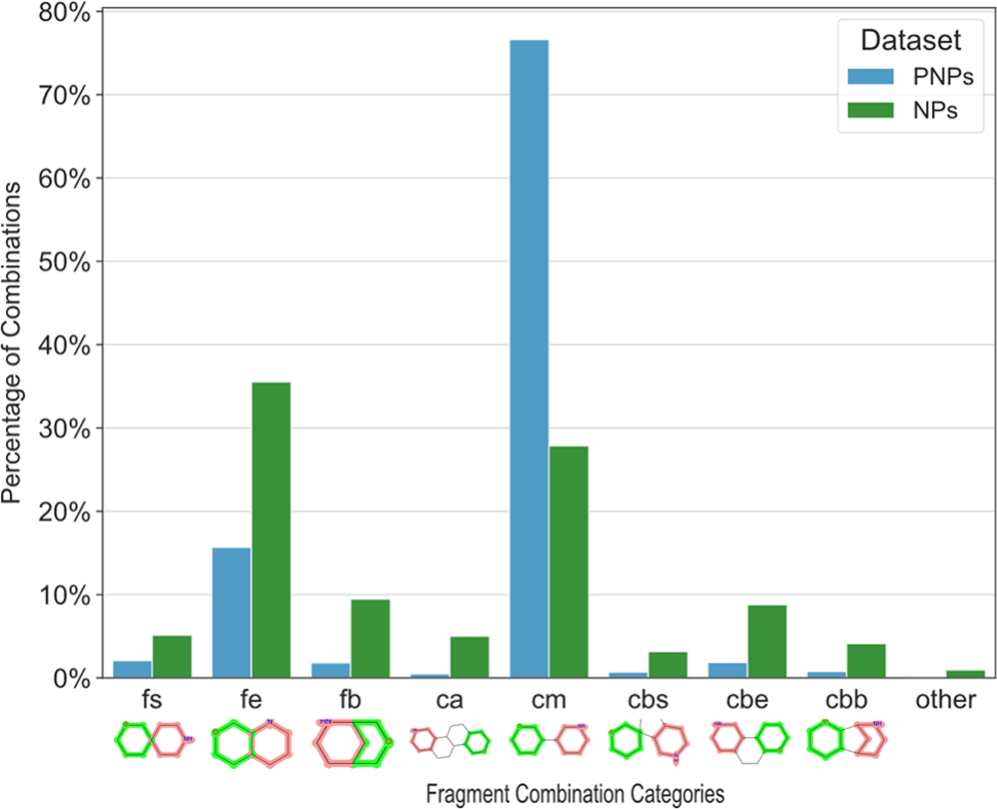
Number of fragment combinations by category. Structures below the bars are simple, manually drawn examples of combinations of two fragments (red and green); **fs**: fusion spiro, **fe**: fusion edge, **fb**: fusion bridged, **ca**: connection annulated, **cm**: connection monopodal, **cbs**: connection bipodal spiro, **cbe**: connection bipodal edge, **cbb**: connection bipodal bridged, and other: aggregation of all other categories with <1% of the total number of combinations.

The 18 fragment combination categories were identified with different frequencies for the two data sets. For NPs, fragments were found to be involved in 78,603 combinations in total, which were divided into eight main classes (Figure 7): fusion edge (**fe**, 35.53% of all combinations), connection monopodal (**cm**, 27.85%), fusion bridged (**fb**, 9.45%), connection bipodal edge (**cbe**, 8.79%), fusion spiro (**fs**, 5.13%), connection annulated (**ca**, 5.01%), connection bipodal bridged (**cbb**, 4.11%), and connection bipodal spiro (**cbs**, 3.17%). For PNPs, the fragment combinations (721,962 in total) most frequently were connection monopodal (**cm**, 76.60%) and fusion edge (**fe**, 15.68%). Other significant combinations observed were fusion spiro (**fs**, 2.07%), connection bipodal edge (**cbe**, 1.85%) and fusion bridged (**fb**, 1.81%), indicating that 98.01% of all PNPs analyzed here were represented by only five different types of combinations.

**Figure 8:**
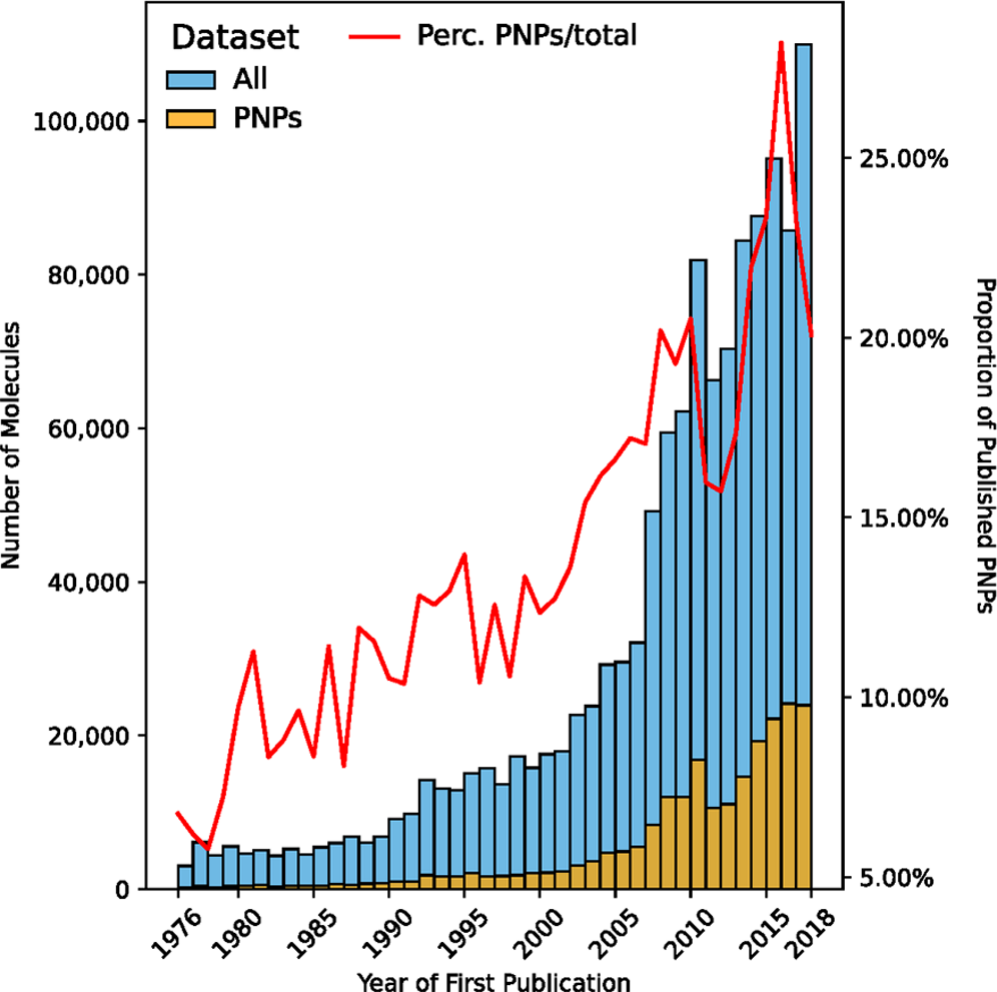
Date of first publication of structures in ChEMBL 26. The data set All refers to all structures in ChEMBL with a document annotation and a valid publication date (1,770,906 records); for PNPs: 224,662 of 344,394 structures (65.23%). The red line indicates the percentage of PNPs of the total of published structures in ChEMBL 26 per year. No data were available after the year 2018. The data for 1974 consisted only of two records and was therefore not considered. For determining the first publication date of compounds, the publication information from duplicate structures filtered out during preparation was considered, when available.

Given this high proportion of PNPs identified in the data set (78.80%), we investigated whether the synthesis and biological analysis of PNPs in the literature was rather a new trend or whether such compounds have historically been reported in comparable numbers. Structures were annotated with the earliest publication dates available, as indicated in ChEMBL (Figure 8). Considering our data set of NP-derived fragments and using the DNP as NP reference, the results clearly indicate that preparation of PNPs has been consistently described over the last 45 years. Remarkably, the percentage of PNPs in published structures per year in ChEMBL increased from 9.81% (±3.08) on average for 1976–2000 to 18.26% (±4.04) for 2000–2018.

**Figure 9:**
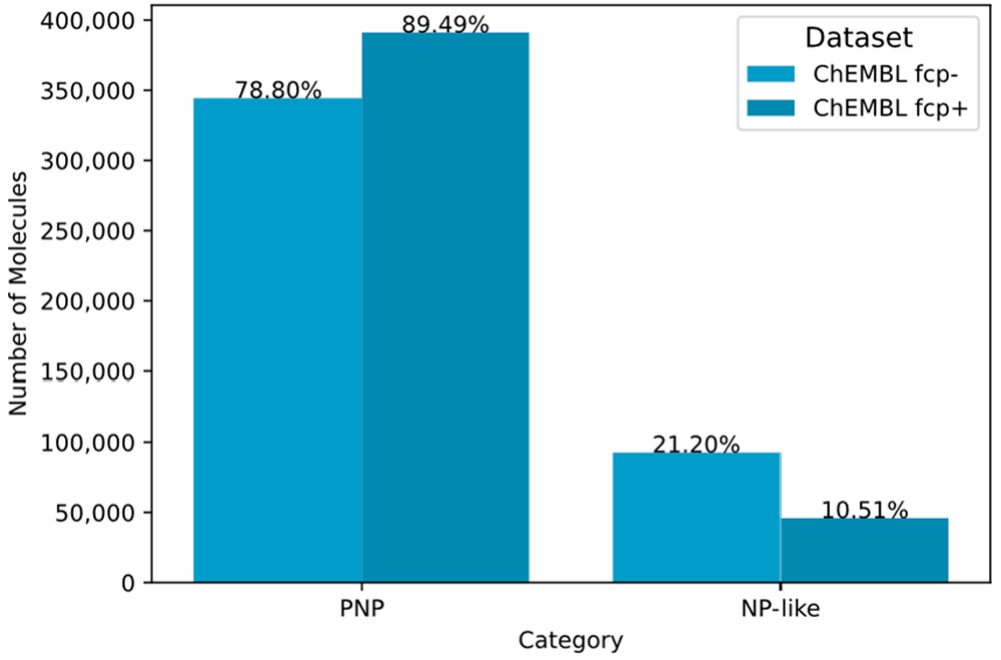
Number of PNPs in ChEMBL with or without considering fragment combination points during PNP annotation (**fcp** ∓).

To investigate whether the same fragments were connected in the same orientation in synthetic compounds and NPs, fragment connection points were considered in addition to the fragment types (nodes) and combination classes (edges), when annotating PNPs. This resulted in a significant increase in the proportion of PNPs, that is, from 78.80% to 89.49% of the remaining synthetic compounds (Figure 9), suggesting that the orientation of fragments varies significantly in combinations across synthetic compounds and NPs.

### PNP Scaffolds

**Figure 10:**
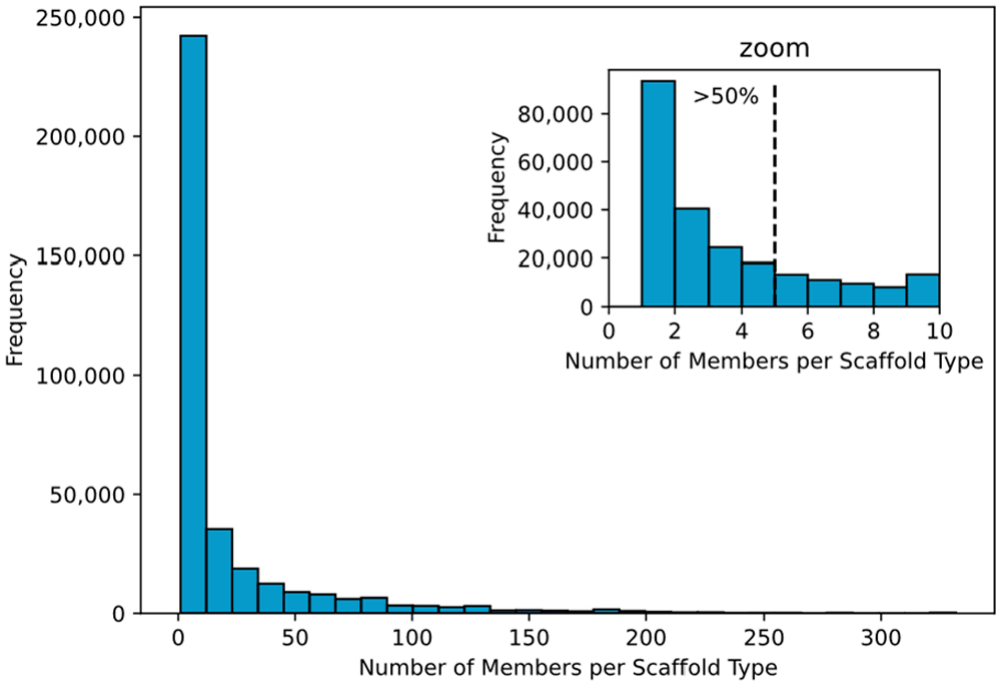
Distribution of the number of members per scaffold type in PNPs. More than half of the data (52.65%) account for scaffold types with 1–5 members.

The fragment connectivity of PNPs, represented as FCGs, was used to define molecular scaffolds. The fragment and combination types together with the fragment connection points information allowed to identify 117,184 unique scaffolds. 25.96% of these scaffolds were represented by only one PNP, whereas more than half of them (52.65%) accounted for small collections of 1–5 compounds and 94.76% contained up to 100 members (Figure 10). For comparison, Murcko scaffolds were extracted from the PNPs as well, amounting to 196,321 unique Murcko scaffolds, which are mostly singletons (73.19%). 91.50% of the unique Murcko scaffold types consisted in collections of up to 10 PNP structures, whereas 98.79% contained up to 100 compounds. These data suggest that the PNPs identified in the ChEMBL database consist of a large number of smaller collections of different compound classes and not of a few large compound libraries.

## Conclusion

We have developed a computational method for preprocessing molecular structures, classifying fragment combinations and characterizing PNPs using a graph approach. To this end, a set of 2000 biologically relevant and diverse NP-derived fragments, previously described by Over et al.,^26^ was used to define the fragment space. During the preparation of the fragments, Murcko scaffolds were extracted. This frequently resulted in the generation of the benzene ring as a fragment, which was found to be overrepresented in ChEMBL. The benzene ring was therefore removed from the fragment pool, yielding a total of 1673 NP fragments.

The Dictionary of Natural Products (DNP) was used to define the chemical space represented by NPs. The 1673 NP fragments were searched within each of the NP structures after preparation. Fragment combinations were classified using the relative position of fragment pairs within the molecules, resulting in 18 different possible categories, which ultimately might be employed to design synthesis targets and efforts. Fragment connectivity was represented as one, or possibly several, alternate graphs of fragments (nodes) and combinations (edges).

The same protocol was applied to the ChEMBL database used to represent synthetic compounds, with the additional step of filtering NPs from the data set using standardized molecule identity (InChI Key) before fragment search. Fragment combination graphs from synthetic compounds were then compared with the graphs obtained previously from the NPs to identify PNPs, that is, synthetic compounds containing NP-derived fragments in combinations that could not be found in any NP structure.

A high percentage of the synthetic compounds remaining at the end of the pipeline matched the PNP criteria (78.80%). These PNPs usually contained 2–4 fragments, involved in 5 main types of combinations (**cm**, **fe**, **fs**, **cbe**, and **fb**). The orientation of the fragments within the combinations was investigated as well, while considering symmetry, by adding another criterion to the comparison of fragment combination graphs. Results indicated that the orientation of fragments varied in synthetic compounds compared to NPs, which led to an increase of the rate of PNPs to 88.98% of synthetic compounds remaining at the end of the pipeline.

Stereochemistry was not considered during during the analysis described above, as the methodology is independent of stereochemical information. The results obtained from the classification of fragment combinations and therefore the annotation of PNPs would not vary with stereochemistry. However, we stress that stereochemistry is an important aspect to be considered in the synthesis of PNPs, since biological activity may vary with configuration. Thus, in the synthesis of PNPs following our analysis, the lack of stereochemical information has to be counterbalanced by the synthesis and biological evaluation of multiple stereoisomers. We note that in our related experimental work, we followed this guideline, see for instance Grigalunas et al.,^34^ Christoforow et al.,^11^ and Liu et al.^35^

These results demonstrate that PNPs, as defined by us, have, in fact, been synthesized for at least 45 years, and it can be concluded that they occur frequently among biologically relevant small molecules. The frequency of their occurrence in biologically relevant compounds is a testimony to their biological relevance and validates the PNP design principle as a successful and actually historically proven general concept for the discovery of new bioactive chemical matter and the exploration of biologically relevant chemical space.

## Data and Software Availability

The npfc package is freely available at https://github.com/mpimp-comas/npfc. The installation guidelines on the repository page describe the installation process with all required dependencies. The initial NP-derived fragments can be downloaded from the Supporting Information of ref (26). The ChEMBL 26 data set is accessible using the link from ref (32). A commercial agreement was necessary for us to use the Dictionary of Natural Products.

## Supporting Information Available

The structures of the set of 1673 NP-derived fragments obtained after preparation in SDF format as well as a molecular grid in PDF, with molecules annotated with fragment connection labels, the statistics applied to investigate the distribution shapes of fragment molecule coverage, and a summary of the results obtained using benzene as a NP-derived fragment (PDF)

## References

[ref1] Newman D. J.; Cragg G. M. Natural Products as Sources of New Drugs over the Nearly Four Decades from 01/1981 to 09/2019. J. Nat. Prod. 2020, 83 (3), 770–803. 10.1021/acs.jnatprod.9b01285. 32162523

[ref2] Cottens S.; Kallen J.; Schuler W.; Sedrani R. Derivation of Rapamycin: Adventures in Natural Product Chemistry. Chimia 2019, 73 (7), 581–590. 10.2533/chimia.2019.581. 31431218

[ref3] Karageorgis G.; Foley D. J.; Laraia L.; Waldmann H. Principle and Design of Pseudo-Natural Products. Nat. Chem. 2020, 12 (3), 227–235. 10.1038/s41557-019-0411-x. 32015480

[ref4] Cremosnik G. S.; Liu J.; Waldmann H. Guided by Evolution: From Biology Oriented Synthesis to Pseudo Natural Products. Nat. Prod. Rep. 2020, 37 (11), 1497–1510. 10.1039/D0NP00015A. 33020792

[ref5] Karageorgis G.; Foley D. J.; Laraia L.; Brakmann S.; Waldmann H. Pseudo Natural Products-Chemical Evolution of Natural Product Structure. Angew. Chem., Int. Ed. 2021, 60, 15705 10.1002/anie.202016575. PMC8360037 33644925

[ref6] Erlanson D. A.; Fesik S. W.; Hubbard R. E.; Jahnke W.; Jhoti H. Twenty Years on: The Impact of Fragments on Drug Discovery. Nat. Rev. Drug Discovery 2016, 15 (9), 605–619. 10.1038/nrd.2016.109. 27417849

[ref7] Ceballos J.; Schwalfenberg M.; Karageorgis G.; Reckzeh E. S.; Sievers S.; Ostermann C.; Pahl A.; Sellstedt M.; Nowacki J.; Carnero Corrales M. A.; Wilke J.; Laraia L.; Tschapalda K.; Metz M.; Sehr D. A.; Brand S.; Winklhofer K.; Janning P.; Ziegler S.; Waldmann H. Synthesis of Indomorphan Pseudo-Natural Product Inhibitors of Glucose Transporters GLUT-1 and −3. Angew. Chem., Int. Ed. 2019, 58 (47), 17016–17025. 10.1002/anie.201909518. PMC6900016 31469221

[ref8] Karageorgis G.; Reckzeh E. S.; Ceballos J.; Schwalfenberg M.; Sievers S.; Ostermann C.; Pahl A.; Ziegler S.; Waldmann H. Chromopynones Are Pseudo Natural Product Glucose Uptake Inhibitors Targeting Glucose Transporters GLUT-1 and −3. Nat. Chem. 2018, 10 (11), 1103–1111. 10.1038/s41557-018-0132-6. 30202104

[ref9] Schneidewind T.; Kapoor S.; Garivet G.; Karageorgis G.; Narayan R.; Vendrell-Navarro G.; Antonchick A. P.; Ziegler S.; Waldmann H. The Pseudo Natural Product Myokinasib Is a Myosin Light Chain Kinase 1 Inhibitor with Unprecedented Chemotype. Cell Chem. Biol. 2019, 26 (4), 512–523. 10.1016/j.chembiol.2018.11.014. 30686759

[ref10] Foley D. J.; Zinken S.; Corkery D.; Laraia L.; Pahl A.; Wu Y.-W.; Waldmann H. Phenotyping Reveals Targets of a Pseudo-Natural-Product Autophagy Inhibitor. Angew. Chem., Int. Ed. 2020, 59 (30), 12470–12476. 10.1002/anie.202000364. PMC7383971 32108411

[ref11] Christoforow A.; Waldmann H.; Wilke J.; Binici A.; Pahl A.; Ostermann C.; Sievers S. Design, Synthesis and Phenotypic Profiling of Pyrano-Furo-Pyridone Pseudo Natural Products. Angew. Chem., Int. Ed. 2019, 58, 14715 10.1002/anie.201907853. PMC7687248 31339620

[ref12] Wu G.; Yu G.; Yu Y.; Yang S.; Duan Z.; Wang W.; Liu Y.; Yu R.; Li J.; Zhu T.; Gu Q.; Li D. Chemoreactive-Inspired Discovery of Influenza A Virus Dual Inhibitor to Block Hemagglutinin-Mediated Adsorption and Membrane Fusion. J. Med. Chem. 2020, 63 (13), 6924–6940. 10.1021/acs.jmedchem.0c00312. 32520560

[ref13] Yuan S.; Yue Y.-L.; Zhang D.-Q.; Zhang J.-Y.; Yu B.; Liu H.-M. Synthesis of New Tetracyclic Benzodiazepine-Fused Isoindolinones Using Recyclable Mesoporous Silica Nanoparticles. Chem. Commun. 2020, 56 (77), 11461–11464. 10.1039/D0CC04875E. 32853306

[ref14] Hert J.; Irwin J. J.; Laggner C.; Keiser M. J.; Shoichet B. K. Quantifying Biogenic Bias in Screening Libraries. Nat. Chem. Biol. 2009, 5 (7), 479–483. 10.1038/nchembio.180. 19483698 PMC2783405

[ref15] Nören-Müller A.; Wilk W.; Saxena K.; Schwalbe H.; Kaiser M.; Waldmann H. Discovery of a New Class of Inhibitors of Mycobacterium Tuberculosis Protein Tyrosine Phosphatase B by Biology-Oriented Synthesis. Angew. Chem., Int. Ed. 2008, 47 (32), 5973–5977. 10.1002/anie.200801566. 18604796

[ref16] de Carne-Carnavalet B.; Krieger J.-P.; Folleas B.; Brayer J.-L.; Demoute J.-P.; Meyer C.; Cossy J. Diastereodivergent Pictet–Spengler Cyclization of Bicyclic N-Acyliminium Ions: Controlling a Quaternary Stereocenter. Eur. J. Org. Chem. 2015, 2015 (6), 1273–1282. 10.1002/ejoc.201403469.

[ref17] Bartlett M. J.; Turner C. A.; Harvey J. E. Pd-Catalyzed Allylic Alkylation Cascade with Dihydropyrans: Regioselective Synthesis of Furo[3,2-c]Pyrans. Org. Lett. 2013, 15 (10), 2430–2433. 10.1021/ol400902d. 23621816

[ref18] Cunha M. R.; Bhardwaj R.; Carrel A. L.; Lindinger S.; Romanin C.; Parise-Filho R.; Hediger M. A.; Reymond J.-L. Natural Product Inspired Optimization of a Selective TRPV6 Calcium Channel Inhibitor. RSC Med. Chem. 2020, 11 (9), 1032–1040. 10.1039/D0MD00145G. 33479695 PMC7513592

[ref19] Reilly S. W.; Griffin S.; Taylor M.; Sahlholm K.; Weng C.-C.; Xu K.; Jacome D. A.; Luedtke R. R.; Mach R. H. Highly Selective Dopamine D3 Receptor Antagonists with Arylated Diazaspiro Alkane Cores. J. Med. Chem. 2017, 60 (23), 9905–9910. 10.1021/acs.jmedchem.7b01248. 29125762 PMC5767125

[ref20] Alkhaibari I.; Raj Kc H.; Alnufaie R.; Gilmore D. F.; Alam M. A. Synthesis of Chimeric Thiazolo-Nootkatone Derivatives as Potent Antimicrobial Agents. ChemMedChem 2021, 16, 2628 10.1002/cmdc.202100230. 33955181 PMC8429137

[ref21] Gaulton A.; Hersey A.; Nowotka M.; Bento A. P.; Chambers J.; Mendez D.; Mutowo P.; Atkinson F.; Bellis L. J.; Cibrián-Uhalte E.; Davies M.; Dedman N.; Karlsson A.; Magariños M. P.; Overington J. P.; Papadatos G.; Smit I.; Leach A. R. The ChEMBL Database in 2017. Nucleic Acids Res. 2017, 45, D945–D954. 10.1093/nar/gkw1074. 27899562 PMC5210557

[ref22] RDKit: Open-source cheminformatics http://www.rdkit.org/ (accessed 2021-03-15).

[ref23] McKinney W. Data Structures for Statistical Computing in Python. Proceedings of the 9th Python in Science Conference (SciPy 2010); Austin, TX, June 28−July 3, 2010; pp 56–61. 10.25080/Majora-92bf1922-00.

[ref24] Hagberg A.; Swart P.; Chult D. S. Exploring Network Structure, Dynamics, and Function Using Networkx; LA-UR-08-05495; LA-UR-08-5495; Los Alamos National Laboratory (LANL), Los Alamos, NM, 2008.

[ref25] Mölder F.; Jablonski K. P.; Letcher B.; Hall M. B.; Tomkins-Tinch C. H.; Sochat V.; Forster J.; Lee S.; Twardziok S. O.; Kanitz A.; Wilm A.; Holtgrewe M.; Rahmann S.; Nahnsen S.; Köster J. Sustainable Data Analysis with Snakemake. F1000Research 2021, 10, 33 10.12688/f1000research.29032.1. 34035898 PMC8114187

[ref26] Over B.; Wetzel S.; Grütter C.; Nakai Y.; Renner S.; Rauh D.; Waldmann H. Natural-Product-Derived Fragments for Fragment-Based Ligand Discovery. Nat. Chem. 2013, 5 (1), 21–28. 10.1038/nchem.1506. 23247173

[ref27] Standardization. MolVS 0.1.1 documentation; https://molvs.readthedocs.io/en/latest/guide/standardize.html (accessed 2020-01-21).

[ref28] pdbeccdutils · master · pdbe/ccdutils; https://github.com/pdbeurope/ccdutils (accessed 2021-10-14).

[ref29] RDKit mailing list - symmetry class; https://sourceforge.net/p/rdkit/mailman/message/27897393/ (accessed 2021-03-11).

[ref30] Dictionary of Natural Products; http://dnp.chemnetbase.com/faces/chemical/ChemicalSearch.xhtml (accessed 2021-02-08).

[ref31] Schaub J.; Zielesny A.; Steinbeck C.; Sorokina M. Too Sweet: Cheminformatics for Deglycosylation in Natural Products. J. Cheminf. 2020, 12 (1), 67 10.1186/s13321-020-00467-y. PMC7641802 33292501

[ref32] Index of /pub/databases/chembl/ChEMBLdb/releases/chembl_26/; http://ftp.ebi.ac.uk/pub/databases/chembl/ChEMBLdb/releases/chembl_26/ (accessed 2021-02-10).

[ref33] Virtanen P.; Gommers R.; Oliphant T. E.; Haberland M.; Reddy T.; Cournapeau D.; Burovski E.; Peterson P.; Weckesser W.; Bright J.; van der Walt S. J.; Brett M.; Wilson J.; Millman K. J.; Mayorov N.; Nelson A. R. J.; Jones E.; Kern R.; Larson E.; Carey C. J.; Polat ı.; Feng Y.; Moore E. W.; VanderPlas J.; Laxalde D.; Perktold J.; Cimrman R.; Henriksen I.; Quintero E. A.; Harris C. R.; Archibald A. M.; Ribeiro A. H.; Pedregosa F.; van Mulbregt P.; et al. SciPy 1.0 Contributors. SciPy 1.0: Fundamental Algorithms for Scientific Computing in Python. Nat. Methods 2020, 17 (3), 261–272. 10.1038/s41592-019-0686-2. 32015543 PMC7056644

[ref34] Grigalunas M.; Burhop A.; Zinken S.; Pahl A.; Gally J.-M.; Wild N.; Mantel Y.; Sievers S.; Foley D. J.; Scheel R.; Strohmann C.; Antonchick A. P.; Waldmann H. Natural Product Fragment Combination to Performance-Diverse Pseudo-Natural Products. Nat. Commun. 2021, 12, 1883 10.1038/s41467-021-22174-4. 33767198 PMC7994817

[ref35] Liu J.; Cremosnik G. S.; Otte F.; Pahl A.; Sievers S.; Strohmann C.; Waldmann H. Design, Synthesis, and Biological Evaluation of Chemically and Biologically Diverse Pyrroquinoline Pseudo Natural Products. Angew. Chem., Int. Ed. 2021, 60 (9), 4648–4656. 10.1002/anie.202013731. PMC7986669 33200868

