# Supplementary material for: Pseudonatural Products Occur Frequently in Biologically Relevant Compounds
Source: J Chem Inf Model. 2021 Oct 20;61(11):5458–68. doi: 10.1021/acs.jcim.1c01084 (PMC8611719; doi:10.1021/acs.jcim.1c01084)
Supplement: Supplementary file 1 — ci1c01084_si_001.pdf [file ci1c01084_si_001.pdf]

Pseudo-Natural Products Occur Frequently in Biologically Relevant Compounds

José-Manuel Gally,† Axel Pahl, ‡ Paul Czodrowski, § Herbert Waldmann*,†,§

†Department of Chemical Biology, Max-Planck-Institute of Molecular Physiology, Otto-Hahn-Straße 11, 44227 Dortmund, Germany,

‡Compound Management and Screening Center, Dortmund, Otto-Hahn-Str. 11, 44227 Dortmund, Germany

§Faculty of Chemistry and Chemical Biology, Technical University Dortmund, Otto-Hahn-Straße 6, 44227 Dortmund, Germany

Supporting Information

TABLE OF CONTENTS

I. List of files included in the Supporting Information S2

II. Manual curation of structural errors in the NP fragments S2

III. Preparation of the datasets S3

IV. Distribution shape analysis of the fragment molecule coverage S13

V. Impact of the benzene fragment on the results S13

VI. Computational time S15

TABLE OF FIGURES

Figure S1 Manual curation of the five NP-derived fragments failing the RDKit conversion. S2
Figure S2. Results of the preparation of the NP-derived fragments dataset......................................S5
Figure S3. Categories of filtered entries in the NP-derived fragments dataset. S6
Figure S4. Results of the preparation of the DNP dataset. S7
Figure S5. Categories of filtered entries in the DNP dataset. S8
Figure S6. Categories of errors raised by entries in the DNP dataset. S9
Figure S7. Results of the preparation of the ChEMBL dataset...S10
Figure S8. Categories of filtered entries in the ChEMBL dataset. S11
Figure S9. Categories of errors raised by entries in the ChEMBL dataset...S12
Figure S10. Probability plots of the observed distributions per dataset. S13
Figure S11. Results for the NPFC workflows including benzene in the NP-fragment. S14

TABLE OF TABLES

Table S1. Possible error cases in NPFC.....S3
Table S2. Filters implemented in NPFC.....S4
Table S3. Number of molecules and entries per step including benzene.....S14

I. List of files included in the Supporting Information

- This document
- The prepared 1,673 NP-derived fragments in SDF format, generated with RDKit
- The prepared 1,673 NP-derived fragments in PDF format, with FCP annotations
- The structures from the manuscript in SMILES format
- A Jupyter notebook describing the distribution shape analysis for figures 6e and 6f.

The NP-derived fragment SDF contains different properties:

- idm: molecule identifier (inherited from the "Cluster" property of the original SDF from Over
- inchikey: the molecule InChI Key, computed with RDKit
- _fcp_labels: the fragment combination point labels enabling the unambiguous orientation of the fragment when symmetry centres are present.
- Num_symmetry_groups: the number of different groups of atoms regrouped within the same symmetry group (i.e. 1a, 1b, etc.)

The npfc package is available at https://github.com/mpimp-comas here additional documentation is available on the package itself (API and overall architecture). The "_fcp_labels" property in the provided SDF is pickled and encoded as base64 strings. Please refer to the npfc.utils.decode_object to convert them back into Python dictionaries.

II. Manual curation of structural errors in the NP fragments

Five structures could not be parsed with RDKit directly using the input SDF. Rather than just discarding them, the structures were fixed using MarvinSketch 19.2.0 (Figure S1).

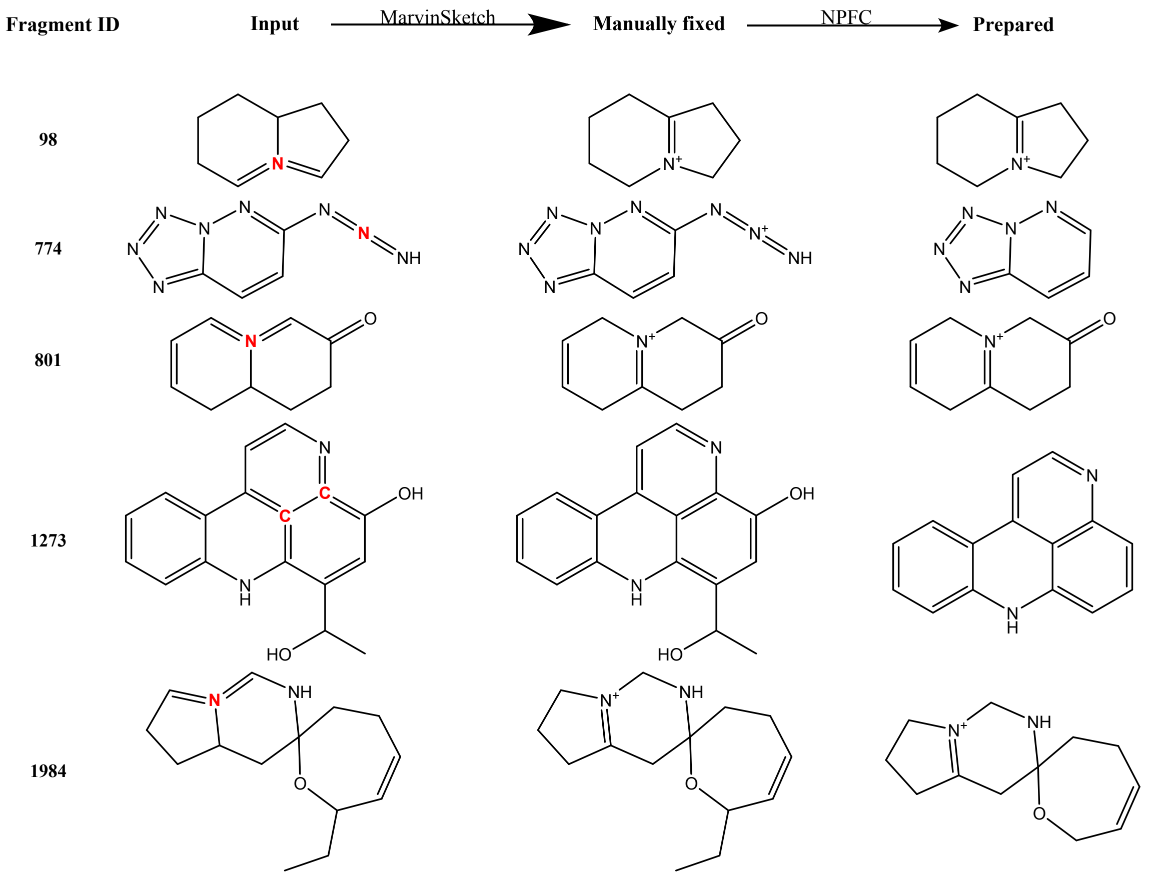

Figure S1 Manual curation of the five NP-derived fragments failing the RDKit conversion. Atoms with valence errors are highlighted in red in the input structures.

III. Preparation of the datasets

The preparation of the datasets consisted of 5 steps:

1. Split the data into chunks (optional)
2. Load the SD File(s) into Pandas DataFrame(s) with RDKit molecules
3. Standardize the structures
4. Removing duplicate entries
5. Depict molecules

During these steps, molecules might fail the process and be discarded (Table S1).

Table S1. Possible error cases in NPFC.

Step	Label	Description
load	cannot_load	The molecule could not be parsed into an RDKit Mol object.
standardize	initiate_mol	The RDKit Mol object could not be initiated from its pickled state.
	disconnect_metal	An error occurred when when disconnecting metal atoms from the RDKit Mol object.

	sanitize	An error occurrend when updating the RDKit Mol object internal properties.
	clear_isotopes	An error occurred when setting the RDKit Mol object atoms to their default isotope.
	normalize	An error occurred when normalizing functional groups of the RDKit Mol object.
	uncharge	An error occurred when removing formal charges from the RDKit Mol object atoms.
	canonicalize	An error occurred when computing the canonical tautomer for the RDKit Mol object.
	clear_stereo	An error occurred when removing the stereochemistry labels from the RDKit Mol object.
	empty_final	The RDKit Mol object was empty at the end of the standardization process.

Similarly, molecules could be filtered out if they did not respect certain criteria (Table S2).

Table S2. Filters implemented in NPFC.

Step	Label	Description
standardize	empty	The RDKit Mol object did not contain any atom.
	num_heavy_atoms	The number of heavy atoms of the RDKit Mol object was outside the expected range. *num_heavy_atoms > 3*
	molecular_weight	The molecular weight (Da) of the RDKit Mol object was outside the expected range. *molecular_weight <= 1000.0*
	num_rings	The number of rings of the RDKit Mol object was outside the expected range. *num_rings > 0*
	elements	The RDKit Mol object contained atoms outside of the expected elements. *elements in H, B, C, N, O, F, P, S, Cl, Br, I.*
	timeout	The standardization process of the RDKit Mol object lasted longer than the timeout limit. *timeout = 10s (per molecule)*
deduplicate	duplicate	The molecule was found to be a duplicate of another, already registered entry. Identity check is performed via InChI Key comparison.

Default values are displayed in italic.

For fragments, only the filters on empty and duplicate structures were enabled, as well as the timeout limit (mandatory).

NP-derived fragments

The 2,000 fragments from Over *et al.* were prepared after five of them were manually curated (see section II). Results are represented by Figure S2 (overall) and Figure S3 (filtered entries).

Natural Products (DNP)

The results of the preparation of the 318,271 records of the DNP dataset are represented by Figure S4 (overall), Figure S5 (filtered entries) and Figure S6 (errors).

Synthetic Compounds (ChEMBL)

The preparation of the 1,941,411 records of the ChEMBL26 dataset are represented by Figure S7 (overall), Figure S8 (filtered entries) and Figure S9 (errors).

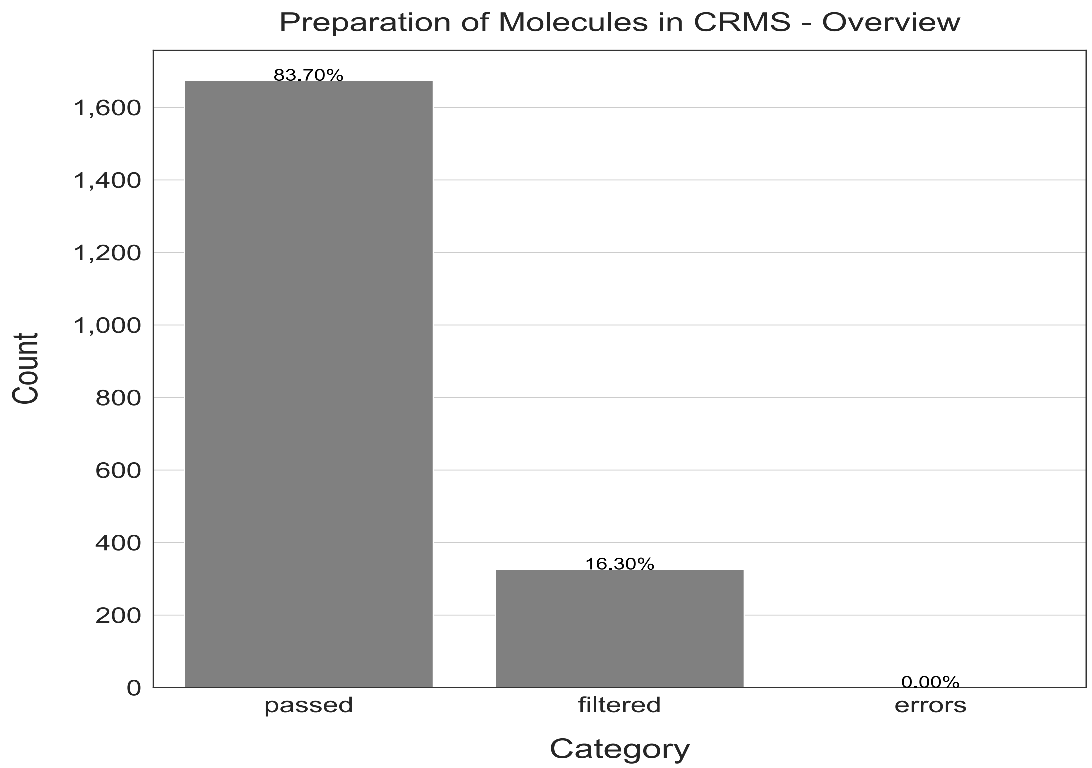

Figure S2. Results of the preparation of the NP-derived fragments dataset. Percentages are relative to the initial number of entries (2,000)

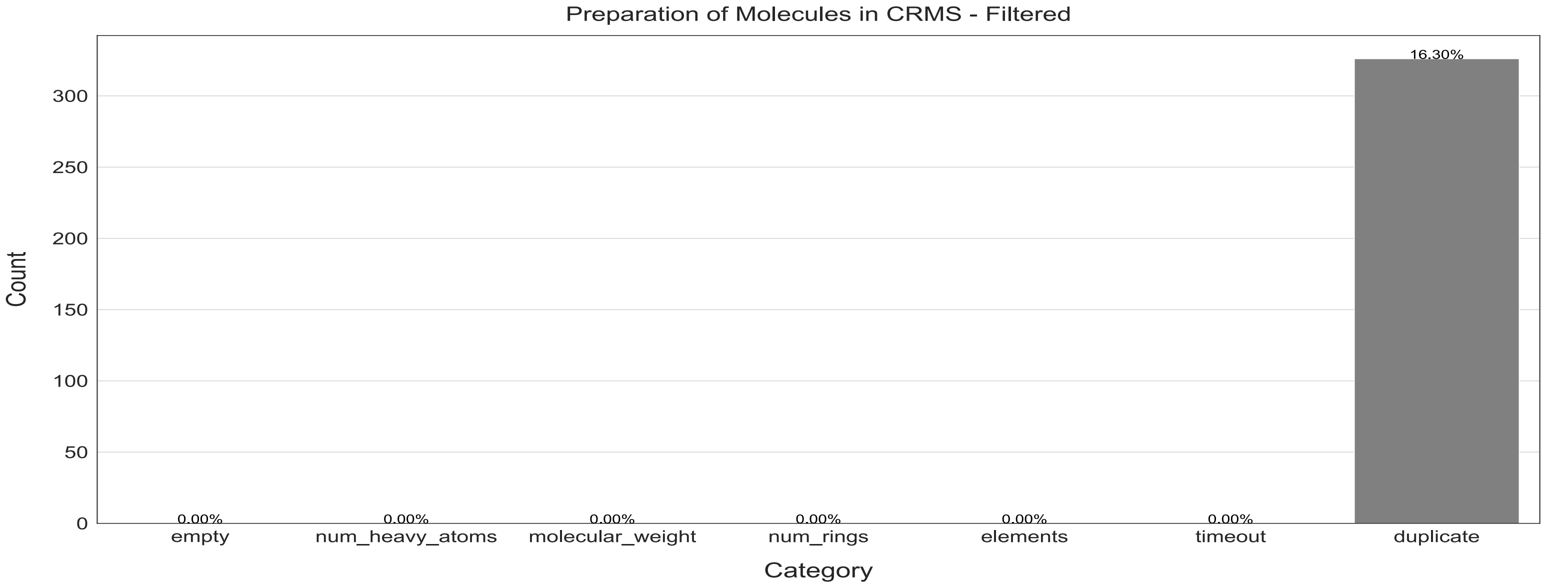

Figure S3. Categories of filtered entries in the NP-derived fragments dataset. Percentages are relative to the initial number of entries (2,000)

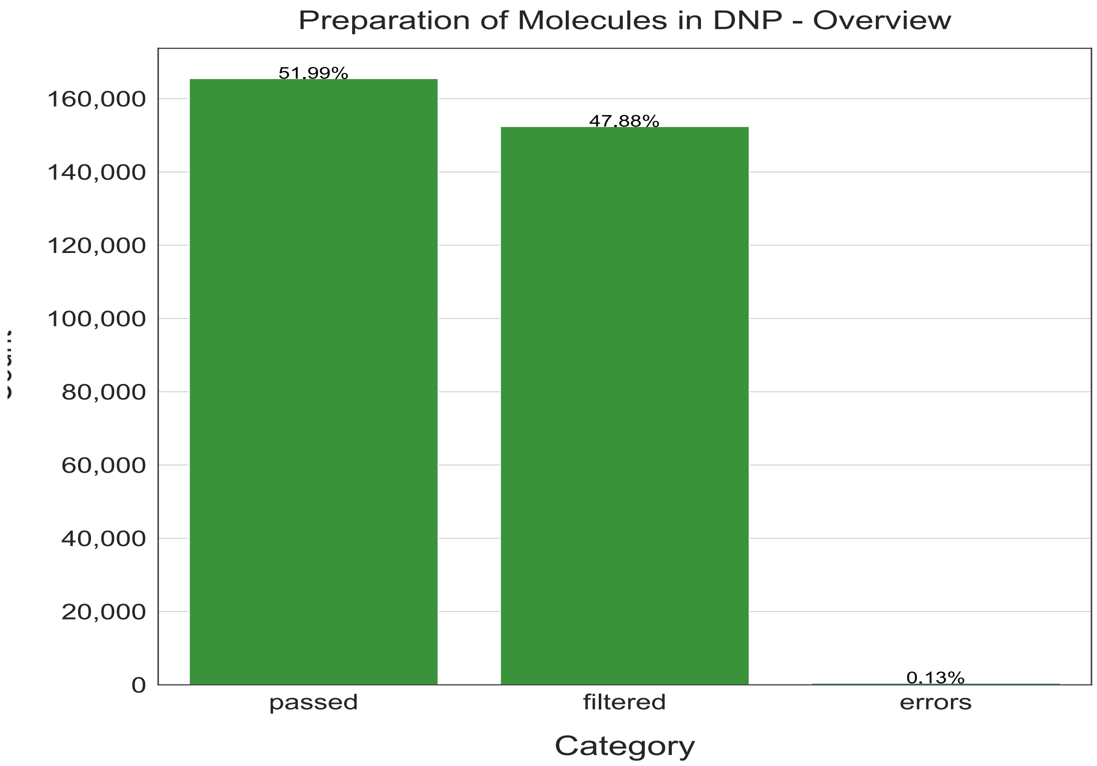

Figure S4. Results of the preparation of the DNP dataset. Percentages are relative to the initial number of entries (318,271)

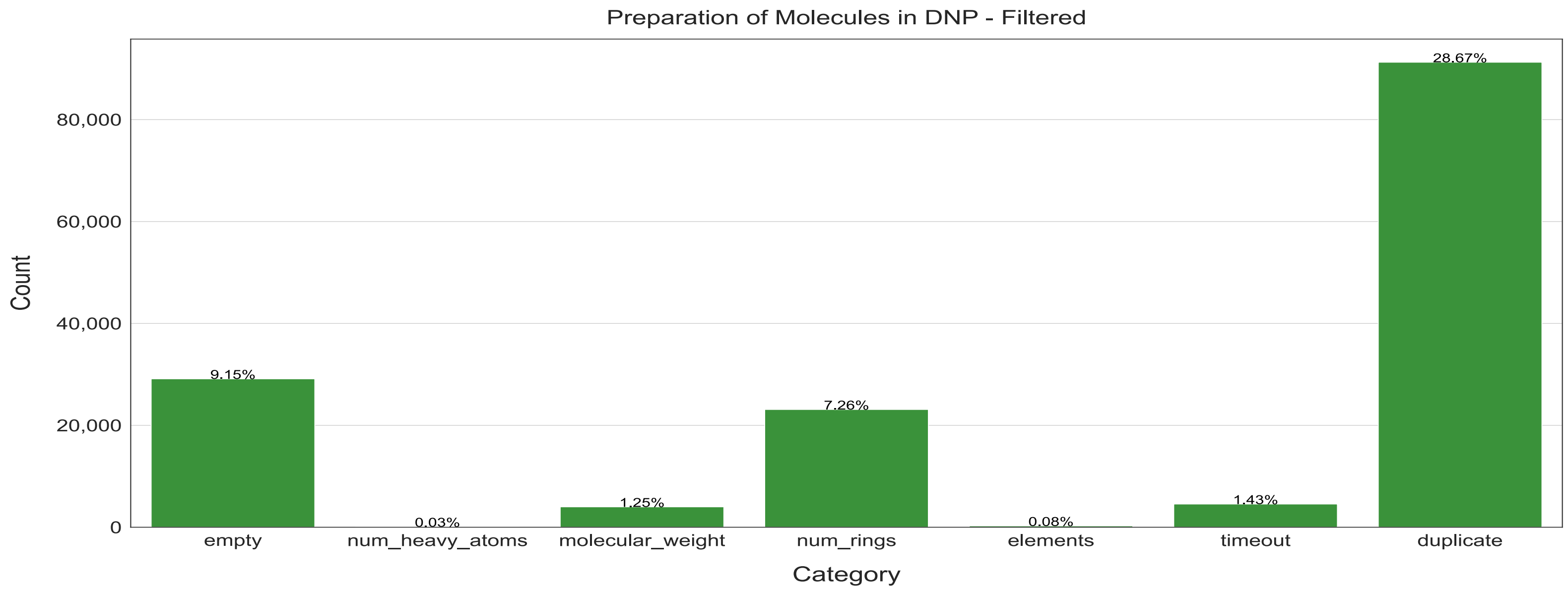

Figure S5. Categories of filtered entries in the DNP dataset. Percentages are relative to the initial number of entries (318,271)

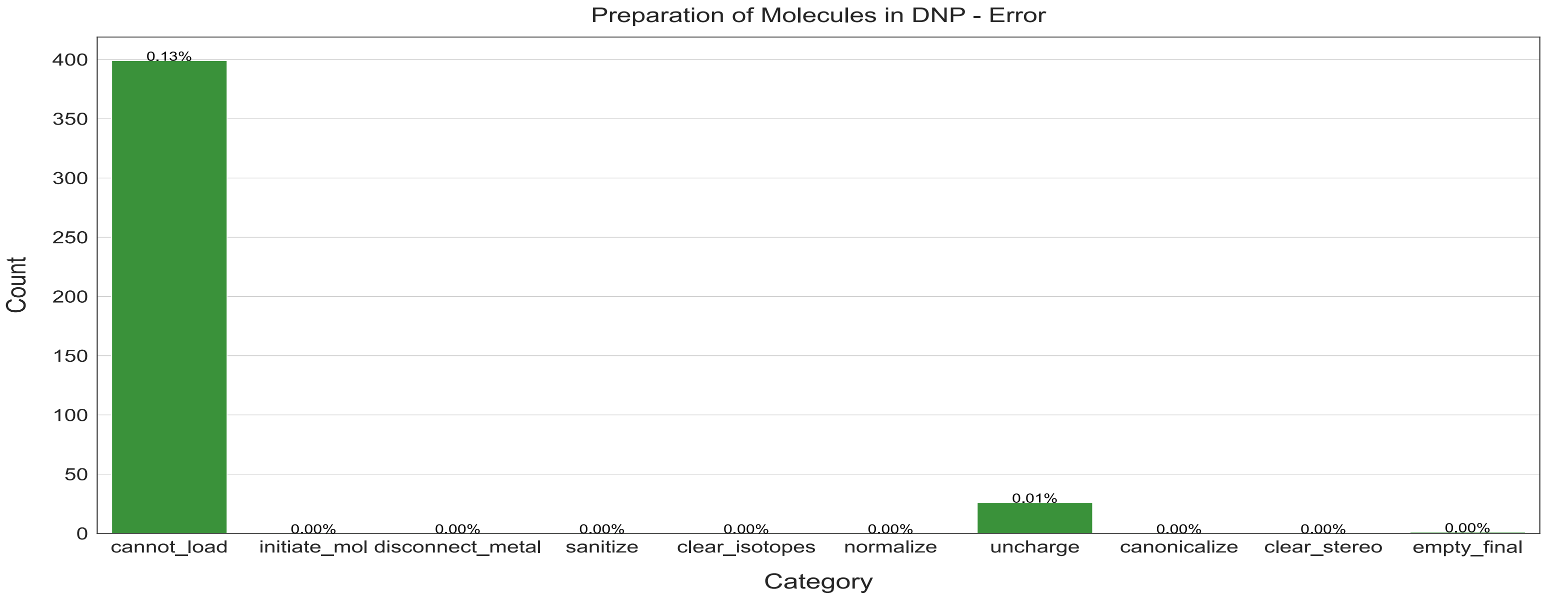

Figure S6. Categories of errors raised by entries in the DNP dataset. Percentages are relative to the initial number of entries (318,271)

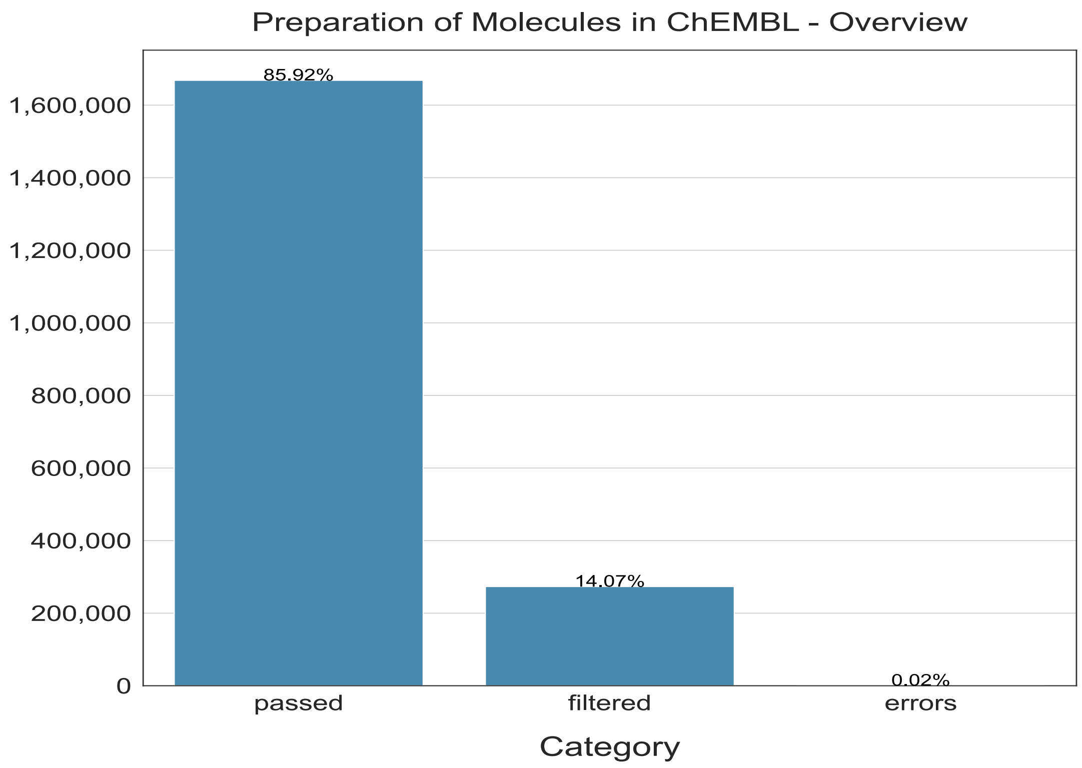

Figure S7. Results of the preparation of the ChEMBL dataset. Percentages are relative to the initial number of entries (1,941,411)

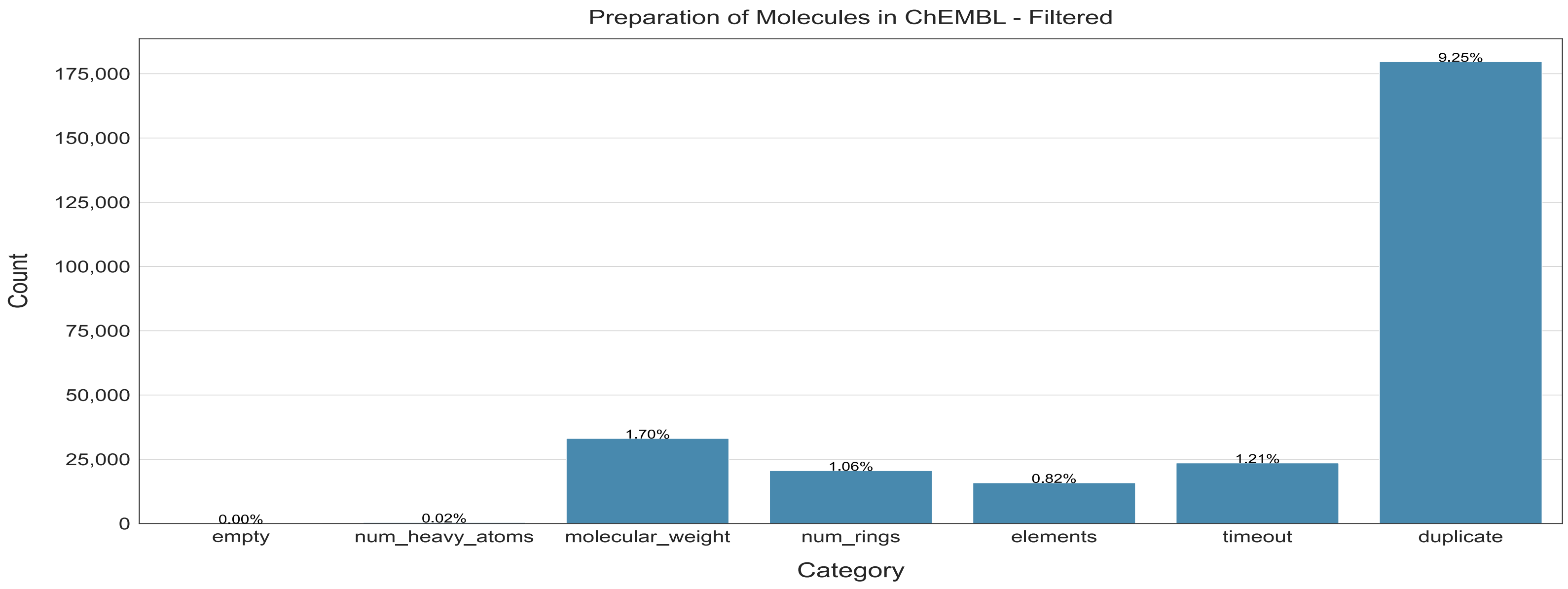

Figure S8. Categories of filtered entries in the ChEMBL dataset. Percentages are relative to the initial number of entries (1,941,411)

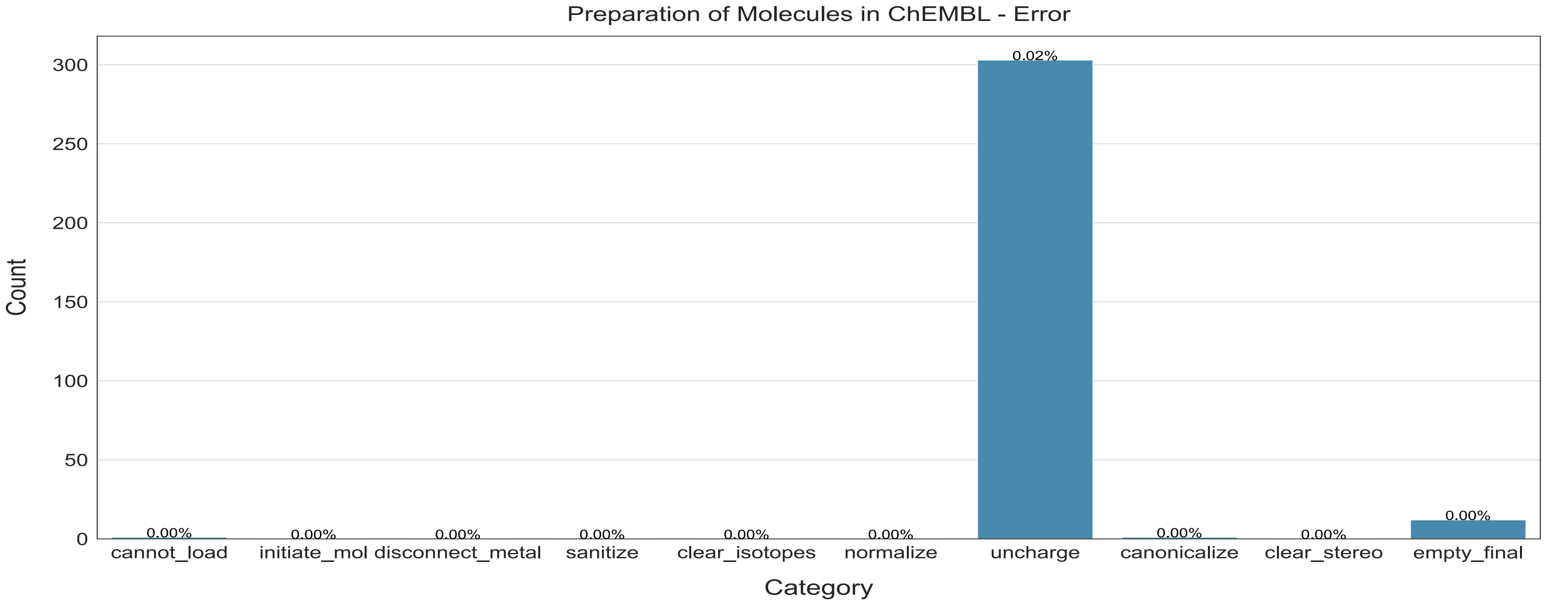

Figure S9. Categories of errors raised by entries in the ChEMBL dataset. Percentages are relative to the initial number of entries (1,941,411)

IV. Distribution shape analysis of the fragment molecule coverage

This section refers to the figures 6e and 6f of the manuscript. Skewness and Kurtosis are already described in the main text and the corresponding code and results are located in the included “distribution_shape_stats.pdf” SI file. Since the distributions for NPs and PNPs when considering side chains (SC+) were bell-shaped, probability plots were computed to check it they could be Gaussian (Figure S10). For comparison, results are presented for both datasets when side chains are not considered as well (SC-). In all cases, the data does not follow a Gaussian distribution.

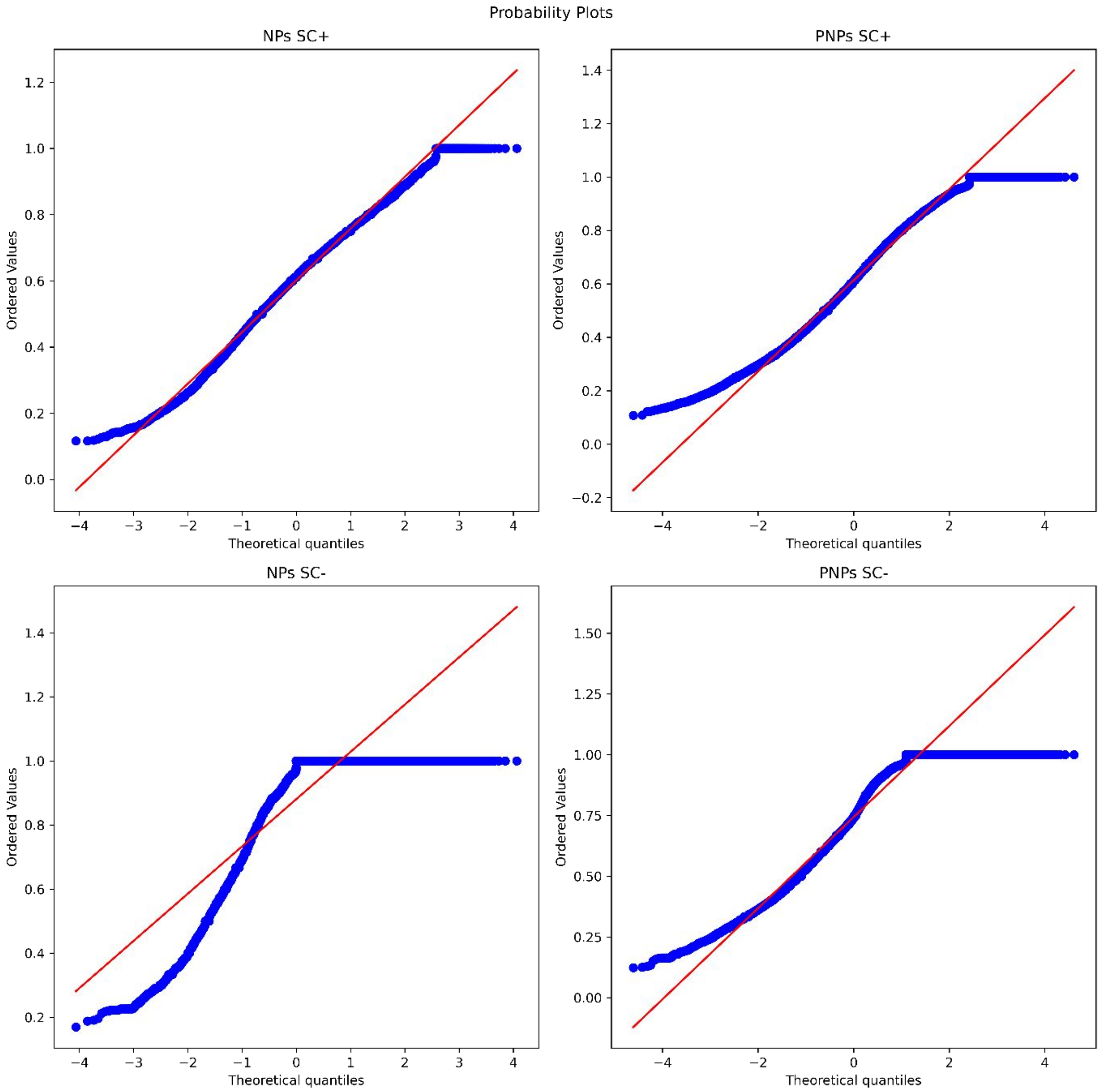

Figure S10. Probability plots of the observed distributions per dataset. The blue line indicates the observed distribution and the red line a Gaussian distribution; NPs: Natural Products, PNPs: pseudo-NPs.

V. Impact of the benzene fragment on the results

The data below describes the results obtained during our first attempt of the NPFC approach, while including the benzene as a Natural Product (NP) fragment. The datasets, software versions and computational resources were the same as used for the main manuscript.

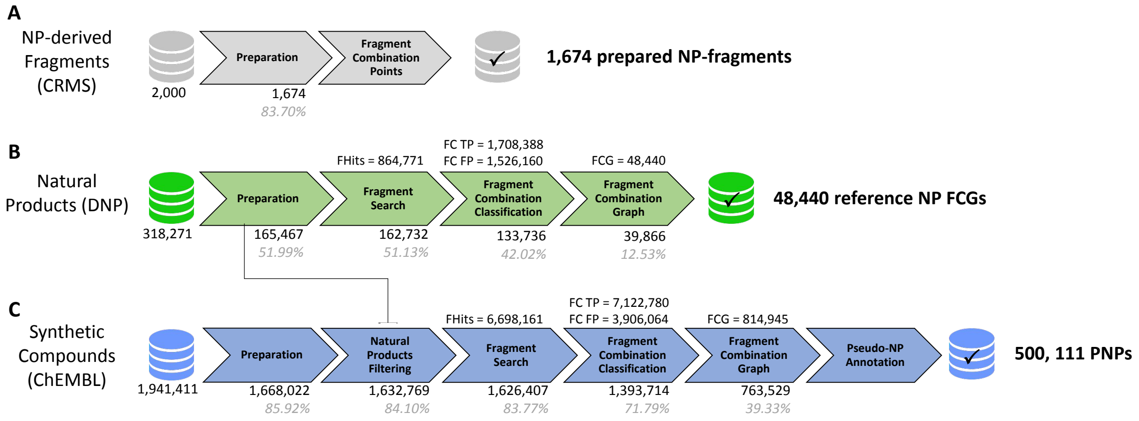

Figure S11. Results for the NPFC workflows including benzene in the NP-fragment. A: results for fragments; B: results for Natural Products; C: results for Synthetic Compounds. The number of remaining molecules at each step is displayed below the tasks, when changes occur. Below it, the percentage of remaining molecules in regards to the initial number is display in grey. Above tasks, the number observed elements is displayed, when different from molecules. Fhits: Fragment Hits; FC: Fragment combinations; TP: True Positive; FP: False Positive; Matches: the number of fragment hits; FCG: Fragment Combination Graphs.

During our first attempt at the NPFC project, we included all fragments obtained from the Over *et al.* dataset. Since the Murcko scaffolds were extracted from the structures (using RDKit), the benzene ring was obtained as an NP-derived fragment on its own and was found to strongly impact the results (Table S3). Indeed, the benzene fragment was found to be part of 37.34% of all remaining NPs at the end of the workflow and represented 26.73% of all fragment combinations at this stage (data not shown). The impact observed for PNPs was even stronger, with the benzene ring found in 63.39% of the structures and representing 44.11% of all fragment combinations.

Table S3. Number of molecules and entries per step including benzene

Step	DNP		ChEMBL	
	**Number of molecules incl. benzene (% of total)**	**Number of entries incl. benzene (% of total)**	**Number of molecules incl. benzene (% of total)**	**Number of entries incl. benzene (% of total)**
**FS**	81,656 (50.18%)	139,140 (16.09%)	1,431,740 (88.03%)	2,537,707 (37.89%)
**FCC**	63,922 (47.80%)	354,169 (10.95%)	1,212,409 (86.99%)	4,918,570 (44.60%)
**FCG**	14,886 (37.34%)	16,920 (34.93%)	530,266 (69.45%	557,805 (68.45%)
**PNP**	N/A		317,005 (63.39%)	333,629 (62.54%)

FS: Fragment Search; FCC: Fragment Combination Classification; FCG: Fragment Combination Graph; PNP: Pseudo-Natural Product. The nature of the entries varies depending on the step, i.e. FS: fragment hits; FCC: fragment combinations; FCG/PNP: fragment combination graphs. Percentages relative to the total of molecules/entries found at each step (with or without benzene) are displayed in parenthesis.

VI. Computational time

Computations were performed on a local cluster of 4 nodes of type Intel® Xeon® Gold 6136, with 12 cores at 3.70 GHZ each (24 threads). Elapsed time was estimated to approx. 90s for the fragments, 40min for the DNP and 4h for the ChEMBL. Cumulated computational time (when considering the running time for all tasks sequentially) was 1min 22s for the fragments, 2 days 11h 32min for the DNP and 16 days 11h 50min for the ChEMBL. Most time-consuming steps were the standardization of the structures (in particular the tautomer canonicalization), the fragment search and to a lower extent, the fragment combination classification (see supporting information for more details). No exhaustive benchmarking was performed.
